# Electroactive Foldamers Endowed with Tetrathiafulvalene Units: From Highly Stable Single Helical Structures to Redox‐Triggered Duplex Formation

**DOI:** 10.1002/asia.202500723

**Published:** 2025-08-13

**Authors:** Soussana Azar, Youssef Aidibi, Lara Faour, Louis Hardoin, C. Elizabeth Killalea, Marie Voltz, Sébastien Goeb, Magali Allain, Ingrid Freuze, Eric Levillain, Christelle Gautier, Marc Sallé, David Canevet

**Affiliations:** ^1^ Univ Angers, CNRS, MOLTECH‐Anjou SFR MATRIX Angers F‐49000 France

**Keywords:** Duplex formation, Electroactive units, Helical structures, Oligoarylamide foldamers, Tetrathiafulvalene, π‐Dimerization

## Abstract

Preparing new smart receptors and materials through controlling foldamer assemblies constitutes an appealing strategy. In this context, the use of a redox input appears as a relevant tool to monitor the self‐assembly process, provided a careful design of well‐chosen electroactive units. Our research group previously showed how the single‐to‐double helix equilibrium of foldamers can be shifted thanks to redox processes. Aiming at generalizing this strategy and rationalizing our findings, we designed a long oligopyridine dicarboxamide strand bearing tetrathiafulvalene (TTF) units, which are connected on the periphery through short amide linkers. This design proved to have a dramatic impact on the supramolecular behavior of the foldamer, preventing the formation of double helices in the neutral state. Using a combination of electrochemical and spectroscopic measurements, we show that duplex formation can be triggered by oxidizing a foldamer that does not form double helices in the neutral state.

## Graphical abstract

Certain foldamers can form multiple helices, which is of interest in materials science, among other fields. By grafting tetrathiafulvalene units onto the ends of a helical oligomer, we report the synthesis and study of a foldamer that shows remarkably high stability in the single‐helical state. Most importantly, we demonstrate that double‐helix formation can be triggered by simple oxidation, enabled by radical ion pairing.

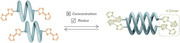

## 1 Introduction

Over the past two decades, helical foldamers have emerged as a new and transformative class of oligomers.^[^ ^1^, ^2^, ^3^ ^]^ These molecules exhibit a remarkable ability to fold into well‐defined structures stabilized by non‐covalent interactions, opening the door to diverse applications in catalysis,^[^ ^4^, ^5^, ^6^, ^7^, ^8^, ^9^ ^]^ host–guest chemistry and sensing,^[^ ^10^, ^11^, ^12^, ^13^ ^]^ molecular machinery,^[^ ^14^, ^15^ ^]^ or chirality‐related applied fields.^[^ ^3^, ^16^ ^]^ Whatever the application under consideration, controlling the geometrical features of helical foldamers appears both fundamental and critical to tuning the physicochemical properties of these molecular architectures. In that, taking advantage of the ability of some foldamer skeletons to form multiple helices^[^ ^17^, ^18^ ^]^ constitutes an opportunity to adjust their properties while avoiding covalent modifications of the backbone. This was notably shown in the contexts of host–guest chemistry^[^ ^19^ ^]^ or chiroptical properties.^[^ ^20^, ^21^ ^]^

**Figure 1:**
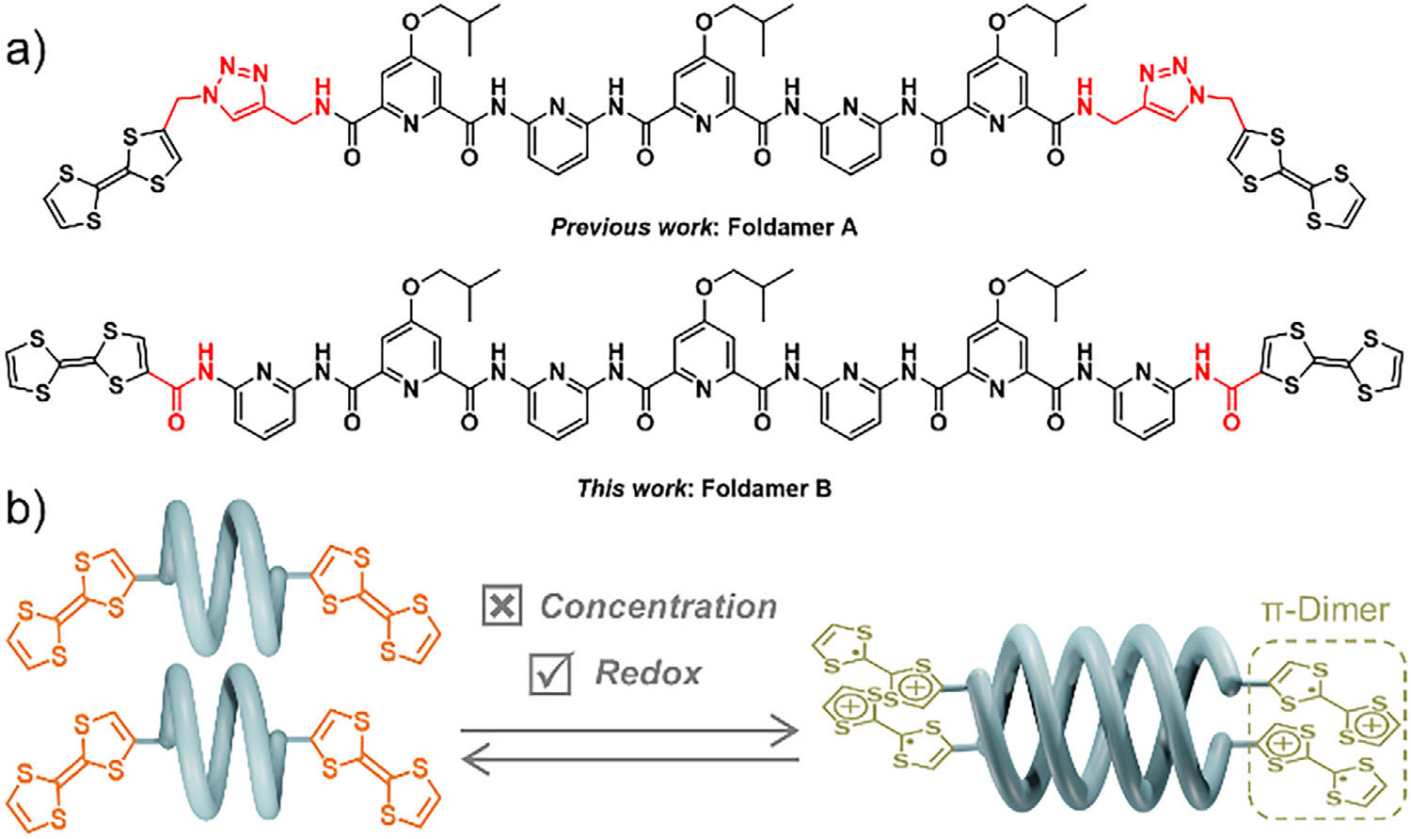
a) Chemical structures of foldamers **A** (five aromatic rings) and **B** (seven aromatic rings). b) Schematic representation of the redox‐triggered duplex formation through π‐dimerization.

Since the hybridization equilibrium of foldamers is governed by solute–solvent and solute–solute interactions, the concentration, temperature, and solvent nature influence the composition of the medium.^[^ ^17^, ^18^ ^]^ While reversible control over this equilibrium has yet to be fully explored, recent breakthroughs highlight the potential for external stimuli to influence this process. The use of photochromes has shown its relevance to govern multiple helix formation in a reversible manner.^[^ ^21^, ^22^ ^]^ Alternatively, redox processes may also constitute an interesting trigger to access both reversibility and fast kinetics, as notably shown with radical cation dimers by Iyoda and coworkers.^[^ ^23^, ^24^, ^25^, ^26^, ^27^, ^28^, ^29^, ^30^, ^31^ ^]^ In a seminal publication,^[^ ^32^ ^]^ we reported that redox stimulations can allow for adjusting the equilibrium constant of hybridization in the case of a tetrathiafulvalene (TTF)‐functionalized foldamer **A** (Figure 1a). In this case, the oxidation of each independent TTF^[^ ^33^ ^]^ redox unit into its respective radical cation state ((**A**)^2(•+)^) proved to favor the double helix formation through radical cation dimerization,^[^ ^34^, ^35^, ^36^, ^37^, ^38^, ^39^, ^40^, ^41^, ^42^ ^]^ with *K*_dim_(**A**)^2(•+)^) = 10^2^ × *K*_dim_(**A**). Aiming at gaining insight on the structural parameters influencing the hybridization ability in the different redox states, as well as expanding the scope of this concept, the target foldamer **B** was designed. In comparison to foldamer **A**, two major structural differences must be highlighted: i) the backbone of foldamer **B** includes two extra pyridyl rings, which is likely to favor double helix formation,^[^ ^43^ ^]^ and ii) a short amide linker connects the helical strand and the redox‐active units, allowing the TTF units to be integrated into the helical backbone and thereby maximizing the impact of their redox states on the hybridization equilibrium. On this basis, we report herein our efforts to synthesize foldamer **B**, its crystallographic structure as a single helix, and its supramolecular behavior in the neutral and the oxidized states. In this way, this study shows how subtle changes in the design of helical foldamers may influence their supramolecular behavior in a dramatic manner, and notably how the redox‐triggering duplex formation is affected (Figure 1b).

## 2 Results and Discussion

### 2.1 Synthesis

**Scheme 1:**
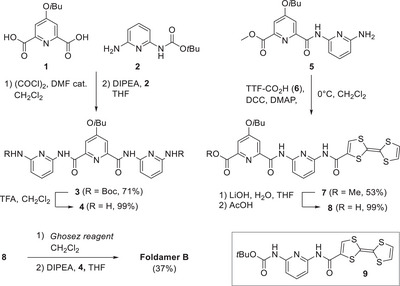
Synthetic scheme followed to isolate foldamer **B** and the chemical structure of reference compound **9**.

The synthetic pathway to foldamer **B** is described in Scheme 1. First, carboxylic acid **1** ^[^ ^32^ ^]^ and amine **2**,^[^ ^44^ ^]^ which were synthesized according to procedures reported in the literature, were coupled in a 71% yield after conversion of **1** into the corresponding acyl chloride. In the presence of trifluoroacetic acid, carbamate **3** was cleaved to afford diamine **4** in a quantitative manner. On the other hand, the redox‐active TTF unit was coupled to both extremities of the foldamer strand through a coupling reaction between amine **5** ^[^ ^21^ ^]^ and carboxyTTF **6**.^[^ ^45^ ^]^ Several coupling reagents were tested, and the best yield was obtained using the DCC/DMAP association, affording the desired product **7** with a 53% yield. Reacting ester **7** with lithium hydroxide and subsequent acidification allowed for isolating carboxylic acid **8** in a quantitative yield. Eventually, target foldamer **B** was obtained with a 37% yield after activation of **8** in the presence of Ghosez reagent and addition–elimination of diamine **4** in the presence of diisopropylethylamine. The structure of the target foldamer **B** was confirmed through standard analytical techniques, and its ^1^H NMR spectrum could be fully assigned through 2D NMR spectroscopy (Figures S1–S6).

Compound **9** (Scheme 1) was prepared as a reference compound for the subsequent characterizations led on foldamer **B**. By analogy to compound **7**, amide **9** was synthesized with a 52% yield through the coupling of carboxyTTF **6** and protected diaminopyridine **2** by using DCC and DMAP.

### 2.2 Solid State Characterization

**Figure 2:**
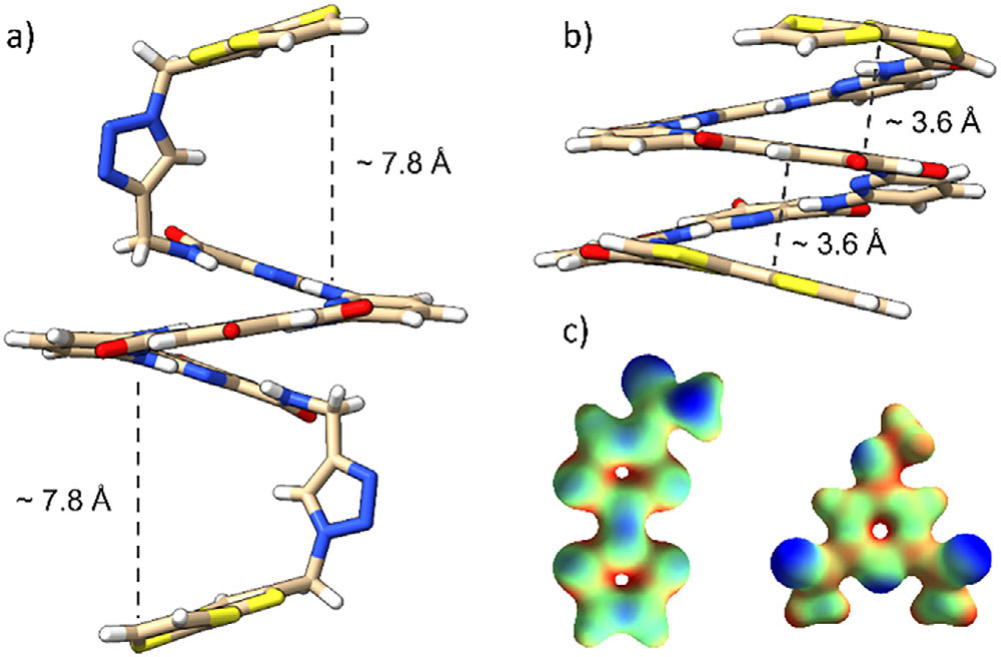
a) X‐ray crystal structures of foldamer **A** (a, CCDC 1891841)[31] and b) **B** (b, CCDC 2430077, crystals grown by slow diffusion of methanol into a chloroform solution**^[^** ^45^ **^]^**). Solvent molecules and isobutyl groups are omitted for clarity. c) Electrostatic potential maps of tetrathiafulvalene‐carboxamide TTF‐CONH_2_ and 4‐methoxypyridine dicarboxamide (DFT, B3LYP, 6–31G*).

Single crystals of foldamer **B** could be grown by slow diffusion of methanol into a chloroform solution. X‐ray diffraction studies confirmed the chemical structure of **B** and its single helical state (Figure 2b).^[^ ^46^ ^]^ The helical conformation appears maintained by a network of intramolecular hydrogen bonds formed between the NH proton of the amide functions and the nitrogen atom of the pyridine units, with bond lengths ranging from 2.18 to 2.38 Å (Figure S7). In addition, pyridine rings also stack on top of each other with the typical distances for aromatic interactions (ca 3.4 Å). Interestingly, the relative arrangement of TTF units strongly differs from the one observed in the case of the previously reported foldamer **A** (Figure 2a). While triazole rings in **A** acted as spacers, adopting a perpendicular orientation with regard to TTF moieties, the connections through short amide linkers in Foldamer **B** allow for the establishment of aromatic interactions between TTF planes and the central pyridyl ring (*d* = 3.6 Å) (Figure 2b). Indeed, it has to be noted that the latter is functionalized with two electron‐withdrawing carboxamide groups, causing the electron deficiency of this heterocycle. Hence, this ring is electronically complementary with TTF units, as confirmed by their respective electrostatic potential maps (Figure 2c). This justifies why this pyridyl ring is sandwiched between both electron‐rich TTF units, as already reported in a foldamer involving pyrene and pyridine dicarboxamide units.^[^ ^47^ ^]^ Interestingly, one will note that no single crystal of a double helical structure could be grown from foldamer **B** despite important efforts, which might be related to the stability of the single helical arrangement.

### 2.3 Supramolecular Behavior in the Neutral State

**Figure 3:**
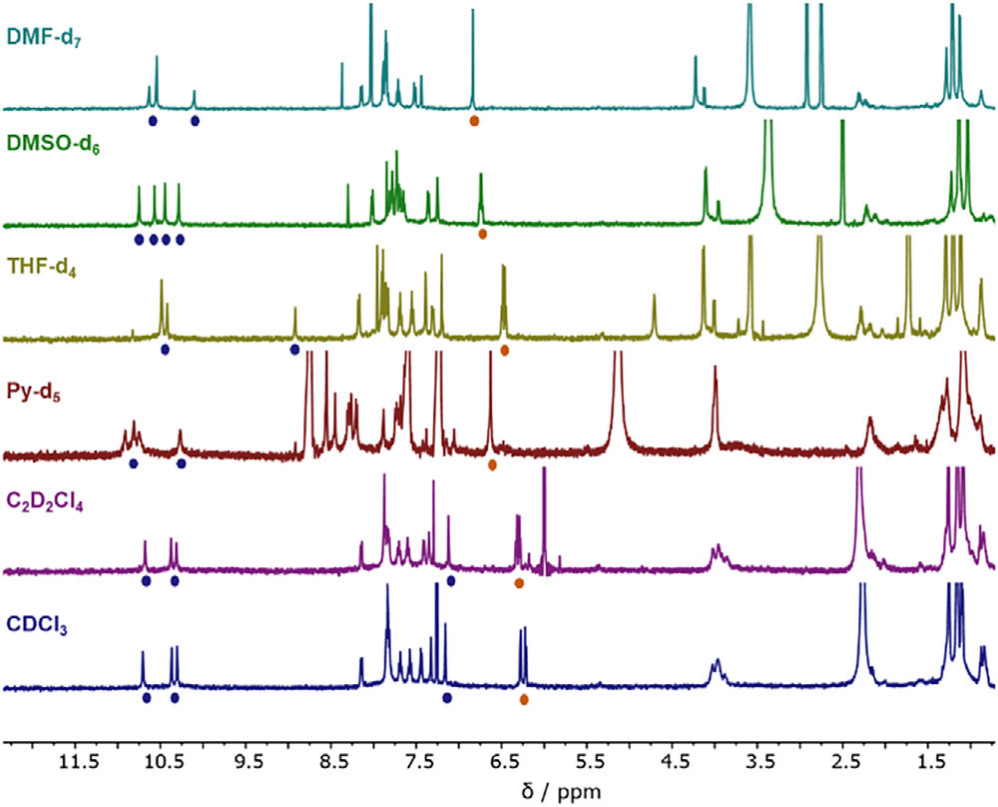
^1^H NMR spectra of foldamer **B** (*C* = 4 mM, 298 K, 500 MHz) in various deuterated solvents. Blue disks indicate NH signals, while orange ones correspond to TTF protons.

The ^1^H NMR study of foldamer **B** was first conducted in deuterated dimethylsulfoxide, which is known to prevent the formation of double helices from oligopyridine dicarboxamide foldamers.^[^ ^43^ ^]^ This allowed for a complete assignment of the signals associated with the single helical state (Figures S1–S6). As mentioned above, the ability of helical foldamers to form multiple helices is inherently linked to solvent‐solute interactions. This prompted us to assess the ability of foldamer **B** to self‐associate in various other deuterated solvents, namely dimethylformamide, tetrahydrofuran, pyridine, tetrachloroethane, and chloroform. These analyses were conducted at room temperature and at a concentration of 4 mM, which guarantees solubility and generally favors the formation of double helices.^[^ ^17^, ^43^, ^48^ ^]^ As illustrated in Figure 3, a single set of signals was systematically observed for amide protons (*δ*_NH_ > 10 ppm). Since these signals are comparable to those detected in DMSO‐d_6_ and since no upfield shielded signals assignable to double helices were observed, it seems reasonable to conclude that foldamer **B** adopts a single helical state, and this whatever the solvent under consideration. This was further confirmed through additional mono‐ and 2D NMR analyses (Figures S8–S15). Measurements carried out at various concentrations (*C* = 0.2 to 5.24 mM, Figure S16) or at different temperatures (273–323 K, Figure S17) never led to the appearance of a second set of signals, which confirms the inability of foldamer **B** to form double helical structures.

### 2.4 Behavior Under Redox Stimulation

**Figure 4:**
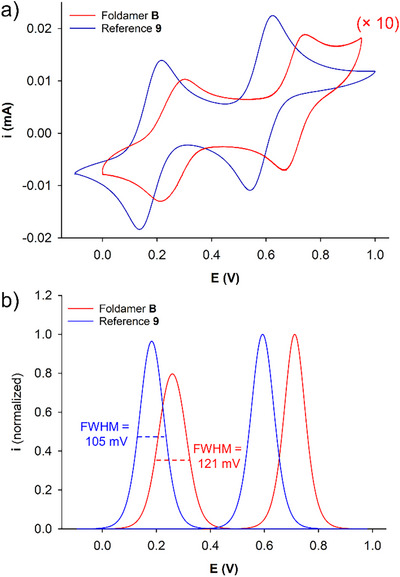
Cyclic voltammograms a) and their deconvolutions b) of foldamer **B** (1.25 × 10^−4^ M, intensity multiplied by 10 to facilitate comparison) and reference **9** (1.25 × 10^−3^ M) (Pt, CHCl_3_/ACN (1/1), *C* = 1.25 × 10^−4^ M, *n*‐Bu_4_NPF_6_ (0.1 M), *v* = 100 mV.s^−1^, and *E* versus Ag/AgNO_3_ (0.01 M)).

Since foldamer **B** does not hybridize in the neutral state, and given that the presence of the electron‐rich TTF units was suspected to contribute to this behavior, evaluating its response to electrochemical stimulation appeared relevant. Foldamer **B** and reference compound **9**, devoid of any oligopyridine helicoidal backbone (Scheme 1), were studied by cyclic voltammetry in chloroform/acetonitrile (1/1 *v/v*) (Figures 4 and S18).^[^ ^49^ ^]^ The corresponding voltammograms (Figure 4a) exhibit two reversible oxidation waves, which correspond to the successive oxidations of each TTF unit into TTF^+•^ and TTF^2+^, respectively.^[^ ^50^ ^]^ One can observe that higher oxidation potentials are required to oxidize the TTF units of foldamer **B** (*E*_1/2_^1^ = 0.26 V, *E*_1/2_^2^ = 0.71 V versus Ag/AgNO_3_) in comparison to reference **9** (*E*_1/2_^1^ = 0.18 V, *E*_1/2_^2^ = 0.59 V versus Ag/AgNO_3_) (Figure 4). Since TTF units are directly linked to a 2,6‐diaminopyridyl fragment in both cases, this difference can only arise from the vicinity of the helical skeleton in foldamer **B**, which patently makes TTF fragments less prone to oxidization. The deconvolution of these voltammograms also appears informative (Figure 4b) since it shows that i) charges are similar in oxidation and reduction, and hence, that redox processes are reversible,^[^ ^51^ ^]^ and ii) foldamer **B** displays a wider first redox wave (full width at half maximum FWHM = 121 mV) in comparison to its second redox wave or to both redox waves of compound **9** (FWHM = 105 mV).^[^ ^52^, ^53^ ^]^ This widening is commonly noticed when two TTF units interact together through a conjugated spacer,^[^ ^54^ ^]^ or when they form through‐space oxidized TTF dimers (Figure 1b).^[^ ^39^, ^42^ ^]^

### 2.5 Spectroelectrochemical Measurements

**Figure 5:**
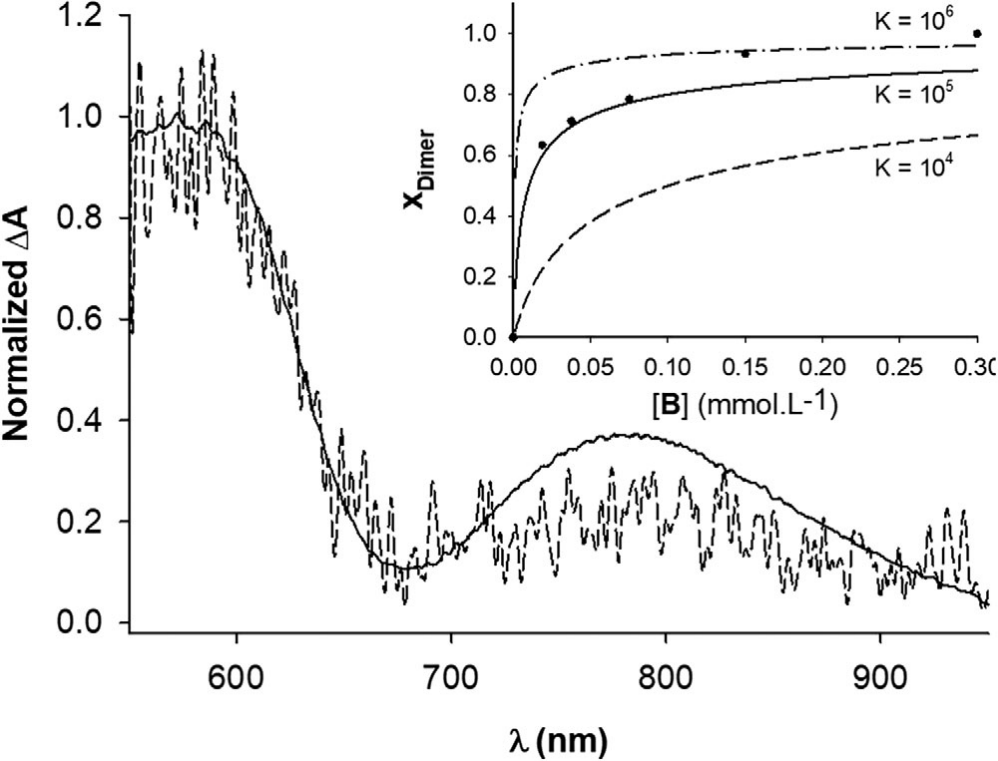
Variations of absorption spectra Δ*A*(λ) = *A*_ox_(λ) − *A*_red_(λ) upon oxidation to the radical cation state for solutions of foldamer **B** at concentrations of 10^−3^ M (solid line) or 1.87 × 10^−5^ M (dashed line). The spectra were extracted from spectroelectrochemical experiments at a potential ensuring oxidation to the TTF^+•^ state (CHCl_3_/CH_3_CN (1/1 v/v), *n*–Bu_4_NPF_6_ (0.1 M) at *v* = 50 mV.s^−1^, under thin layer conditions). Absorbance values are normalized at 580 nm. Inset: evolution of the molar fraction of radical cation dimers as a function of the total concentration of **B**. The molar fraction of dimer was calculated assuming full conversion to radical cation dimers at 0.6 mmol.L^−1^. Curves correspond to theoretical isotherms and dots to experimental values.

To determine whether the broadening of the first redox wave is associated with intra‐ or intermolecular processes, spectroelectrochemical measurements were performed on foldamer **B** at relatively high and low concentrations (10^−3^ M and 1.87 × 10^−5^ M, respectively). By slowly varying the redox potential applied on a thin layer, the variation of absorbance Δ*A*(λ) = *A*_ox_(λ) − *A*_red_(λ) between 550 and 950 nm was recorded at a potential ensuring the oxidation of the tetrathiafulvalene units to their radical cation state (Figure 5). These measurements reveal the appearance of an absorption band centered at *λ* = 580 nm, a characteristic feature of such radical cations.^[^ ^55^, ^56^ ^]^ In addition, a broad absorption band appeared at a higher wavelength (*λ* = 790 nm). 2D graphs extracted at 0.65 V (Figure 5) confirm that this band persists, even at concentrations as low as a 10^−5^ M range. The latter observation indicates the formation of radical cation dimers^[^ ^57^ ^]^ with relatively tight intermolecular contacts. This conclusion was further confirmed by the nonlinear relationship of the molar fraction of dimer plotted as a function of the foldamer concentration (Figure 5, inset). While the fitting attempts did not converge, simulations (see inset) indicate that the order of magnitude of $K_{\dim}^{\mathrm{ox}}$ is about 10^5^. This appears in sharp contrast with most observations reported in the literature regarding oxidized TTF dimers,^[^ ^39^, ^42^ ^]^ for which π‐dimers are most often not observed in such competing solvents at such low concentrations and at room temperature, because the corresponding equilibrium constants are generally small (about 100).

**Figure 6:**
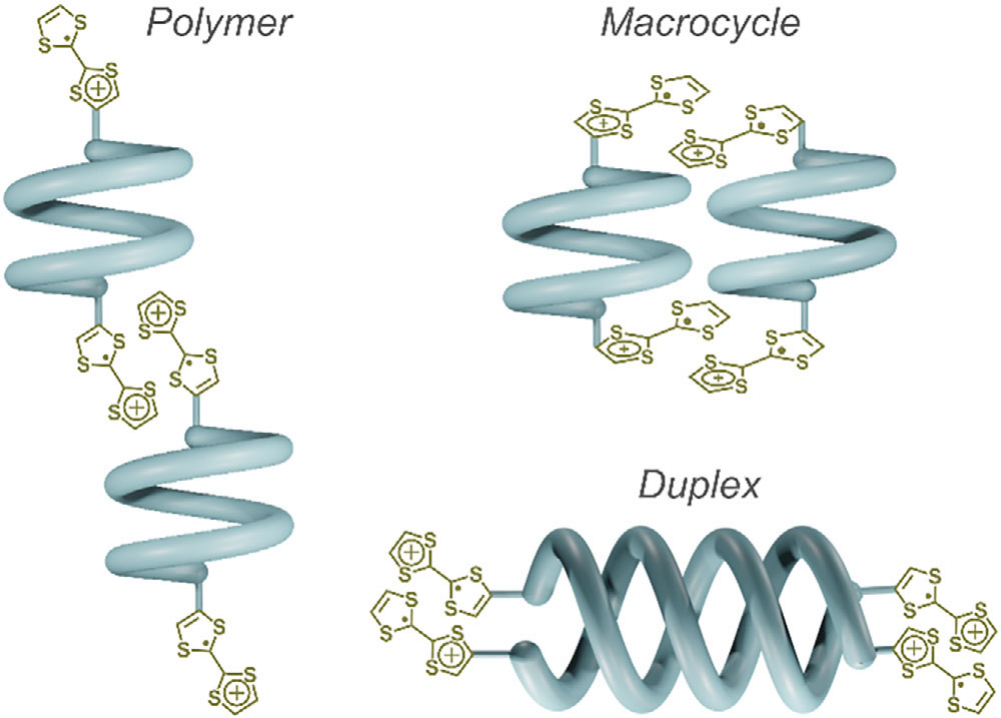
Possible supramolecular arrangements of foldamer **B** after oxidation of TTF units.

In this context, three plausible arrangements can be envisioned: the growth of a supramolecular polymer, the formation of a macrocycle, or the generation of double helices (Figure 6).

To get insight into the possible formation of a supramolecular polymer (Figure 6, left), spectroelectrochemical analyses were performed on reference compound **9**, which, given its structure, cannot form macrocycles (Figure 6, middle) or duplexes (Figure 6, right). This was performed by using the same experimental conditions as those applied for the analysis of foldamer **B**. The corresponding spectroelectrochemical studies revealed that the interactions between TTF radical cations only occur at high concentrations (>7.5 mM—Figure S20, left). Furthermore, by comparing the spectroelectrochemical data of foldamer **B** at 0.3 mM to that of reference compound **9** at 0.6 mM (Figures 5 and S20), one can clearly conclude that the concentration of π‐dimers is definitely higher in the case of foldamer **B**. Moreover, from a quantitative point of view, the evolution of the absorption as a function of log *C* demonstrates that foldamer **B** forms π‐dimers at concentrations that are about three orders of magnitude lower than reference **9** ($K_{\dim}^{\mathrm{ox}}$ (**9**) ≈230, Figure S21). These results indicate that the foldamer skeleton favors the formation of π‐dimers and excludes the supramolecular polymerization of foldamer **B**. Then, considering 1) the structure of foldamer **B** (see X‐ray structure), the network of hydrogen bonds and aromatic interactions that stabilize its helical conformation and, the relative orientation of TTF units, as well as 2) the fact that the well‐preorganized TTF‐substituted systems described by Zhang–Ting Li and coworkers (see Scheme S1) solely display a dimerization constant of ca. 100 in a similar solvent system,^[^ ^37^ ^]^ we consider the formation of supramolecular macrocycles very unlikely. Consequently, the oxidation‐triggered double helix formation of foldamer **B** into duplexes appears logical and is coherent with the formation of π‐dimers at very weak concentrations and in the absence of any additive known for stabilizing these supramolecular assemblies.^[^ ^58^ ^]^ Analyzing the composition of the medium by mass spectrometry after chemical oxidation with methylphenothiazinium tetrafluoroborate further confirmed the formation of foldamer duplexes when TTF units are in the radical cation states (Figure S22). Thereby, the oxidation of TTF units appears as a potentially effective approach to control the stability of the single helical structures. Through oxidation, the properties of TTF units (charge, electron density) evolve, which manifestly leads to the weakening of intramolecular non‐covalent bonds between TTF and the neighboring units within the foldamer framework. This change in interactions involving the terminal TTF units, therefore, allows the usual interactions between two oligopyridine dicarboxamide strands to take place, giving rise to a duplex structure, which is further stabilized through radical cation dimerization.

## 3 Conclusion

The field of stimuli‐responsive foldamers based on redox stimulations is still in its early stages of development. Throughout this work, we synthesized a new oligomer derived from the oligopyridine dicarboxamide skeleton and endowed with tetrathiafulvalene units through amide linkers. Our synthetic efforts allowed the successful preparation of the target redox‐active foldamer in thirteen steps. The studies conducted in solution and in the neutral state highlight the high stability of the single helical arrangement and show its inability to form duplexes. This was also supported in the solid state by a crystallographic study, which highlights short intramolecular contacts between TTF units and the oligoarylamide skeleton. These results evidence the extent to which the nature of the linker constitutes an important parameter that affects the overall behavior of a foldamer functionalized with electroactive units. Eventually, taking advantage of the redox properties of tetrathiafulvalene, we have demonstrated through spectroelectrochemical measurements that its oxidation to the radical cation state allows for observing the duplex formation at particularly low concentrations. To our knowledge, foldamer **B** constitutes the first example of a “redox‐triggered duplex formation”, and this without any alteration of the medium composition. This result constitutes a particularly appealing milestone for the development of stimuli‐responsive materials based on foldamers.

## Conflict of Interests

The authors declare no conflict of interest.

## Supporting Information

Additional figures and experimental details are given within the Supporting Information file.

## Supporting information



Supporting Information
