# Supplementary material for: Electroactive Foldamers Endowed with Tetrathiafulvalene Units: From Highly Stable Single Helical Structures to Redox‐Triggered Duplex Formation
Source: Chem Asian J. 2025 Aug 13;20(19):e00723. doi: 10.1002/asia.202500723 (PMC12498192; doi:10.1002/asia.202500723)
Supplement: Supplementary file 1 — Supporting Information [file ASIA-20-e00723-s001.docx]

**Electroactive Foldamers Endowed with Tetrathiafulvalene units: From Highly Stable Single Helical Structures to Redox-triggered Duplex Formation**

Soussana Azar, Youssef Aidibi,^‡^ Lara Faour,^‡^ Louis Hardoin, Catherine E. Killalea, Marie Voltz, Magali Allain, Ingrid Freuze, Sébastien Goeb, Eric Levillain, Christelle Gautier,* Marc Sallé,* David Canevet*

^‡^ *Equal contributions from both authors.*

Dr S. Azar, Dr Y. Aidibi, Dr L. Faour, L. Hardoin, Dr C. E. Killalea, M. Voltz, Dr S. Goeb, Dr. M. Allain, Dr I. Freuze, Dr E. Levillain, Dr C. Gautier,* Pr M. Sallé,* Pr D. Canevet*

Univ Angers, CNRS, MOLTECH-Anjou, SFR MATRIX, Angers F-49000, France.

E-mail: [christelle.gautier@univ-angers.fr](mailto:christelle.gautier@univ-angers.fr), [marc.salle@univ-angers.fr](mailto:marc.salle@univ-angers.fr), [david.canevet@univ-angers.fr](mailto:david.canevet@univ-angers.fr)

**Supporting information**

**Experimental details**

All reagents and chemicals from commercial sources were used without further purification. Solvents were dried according to standard procedures (Sodium/benzophenone for tetrahydrofuran and diethyl ether, CaH_2_ for dichloromethane, acetonitrile, triethylamine and *N,N*-diisopropylethylamine, P_2_O_5_ for chloroform).

Column chromatography was performed with analytical-grade solvents using Aldrich silica gel (technical grade, pore size 60 Å, 230-400 mesh particle size). Flexible plates ALUGRAM® Xtra SIL G UV254 from MACHEREY-NAGEL were used for TLC.

**Recycling high performance liquid chromatography**

Crude compounds were solubilized in HPLC grade chloroform (stabilized with ethanol). Prior to injection, the solution was filtered through a 0.45 µm PTFE filter (VWR 25mm syringe filter w/ 0.45 µm membrane). Purification was performed on a LC-9160NEXT system from the Japan Analytical Industry Co., Ltd. (JAI) equipped with coupled UV-vis 4Ch NEXT and RI-700 II detectors at room temperature through a set of two JAIGEL-2H and 2.5H columns at an elution rate of 10 mL.min^-1^.

Compounds were detected by UV irradiation (Bioblock Scientific) or staining with iodine, unless otherwise stated.

**NMR Spectroscopy**

NMR spectra were recorded on a Bruker AVANCE III 300 (^1^H, 300 MHz; ^13^C, 75 MHz) or a Bruker AVANCE DRX500 (^1^H, 500 MHz; ^13^C, 125 MHz). Chemical shifts are given in parts per million (ppm) relative to TMS and coupling constants J in Hertz (Hz).

**High Resolution Mass Spectrometry** (HRMS) measurements were performed with a JEOL JMS-700 B/E. To detect the oxidized duplex, a degassed solution of foldamer **B** (3 mM) was treated with one equivalent of phenothiazinium tetrafluoroborate (solvent: CH_2_Cl_2_/CH_3_CN (2/8 *v*/*v*)) and the corresponding solution was analysed by ESI Q-TOF mass spectrometry on a Waters Xevo® G2-XS QTOF equipment.

**Cyclic voltammetry** experiments were carried out on a potentiostat Bio–Logic SP–300. Tetrabutylammonium hexafluorophosphate (0.1 M) was used as supporting electrolyte. The cell was equipped with three electrodes: a platinum working electrode (Ø = 2 mm), a platinum wire as auxiliary electrode and a silver/silver nitrate (0.01 M, CH_3_CN, *n*-Bu_4_NPF_6_) reference electrode. The potentials are given with respect to the Ag/AgNO_3_ redox couple.

**Spectroelectrochemistry** measurements were performed by Dr Christelle Gautier (MOLTECH–Anjou) and were carried out in direct reflecting mode on the working electrode (e.g. Pt) with a homemade bench, developed by Dr Eric Levillain and Dr Olivier Alévêque, composed of different Princeton Instruments modules (light sources, fibers, monochromators, spectroscopy camera, and software). The connection between the light source, the cell and the spectrophotometer is ensured through a “Y–shaped” optical fiber bundle: 18 fibers guide the light to the cell, and 19 fibers collect the reflected light from the cell to visible (320‐1080 nm/maximum acquisition frequency 2 MHz) and IR (900‐1700 nm / maximum acquisition frequency 8 MHz) CCD detectors. The sensitivity of the spectroscopic measurement (< 3 e− at 100 kHz and < 13 e− at 2 MHz between 320 and 1080 nm; 400 e− (high gain) and 5000 electrons (low gain) between 900 nm and 1700 nm) allows performing a spectroelectrochemistry experiment under the usual conditions of electrochemistry.

**X-Ray Diffraction**: Crystal data were collected at 150 K on a Rigaku Oxford Diffraction SuperNova diffractometer equipped with an Atlas CCD detector and micro-focus Cu-K_α_ radiation (λ = 1.54184 Å). The structure was solved by dual-space algorithm and refined on F^2^ by full matrix least-squares techniques using SHELX package (G.M. Sheldrick, ShelXT-2018/2, ShelXL-2019/3). All non-hydrogen atoms were refined anisotropically and the H atoms were included in the calculation without refinement. Absorption was corrected with gaussian technique by using CrysAlisPro program (CrysAlisPro, Rigaku Oxford Diffraction, V1.171.40.45a, 2019). Deposition Number(s) 2430077 for **B** contain(s) the supplementary crystallographic data for this paper. These data are provided free of charge by the joint Cambridge Crystallographic Data Centre and Fachinformationszentrum Karlsruhe Access Structures service.

Crystallographic data for **B**: C_139_H_137_Cl_6_N_30_O_27.5_S_16_, M = 3393.44, yellow plate, 0.300 x 0.131 x 0.027 mm^3^, triclinic, space group *P*-1, a = 14.6163(8) Å, b = 15.216(1) Å, c = 19.646(1) Å, α = 72.163(5)°, β = 75.972(5)°, γ = 69.475(5)°, V = 3850.4(4) Å^3^, Z = 1, ρcalc = 1.463 g/cm^3^, μ = 3.716 mm^-1^, F(000) = 1759, θmin = 3.198 °, θmax = 77.208°, 33208 reflections collected, 15285 unique (R_int_ = 0.0751), parameters / restraints = 1022 / 16, R1 = 0.0780 and wR2 = 0.2020 using 9220 reflections with I>2σ(I), R1 = 0.1245 and wR2 = 0.2283 using all data, GOF = 1.034, -0.822 < Δρ < 0.766 e.Å^-3^.

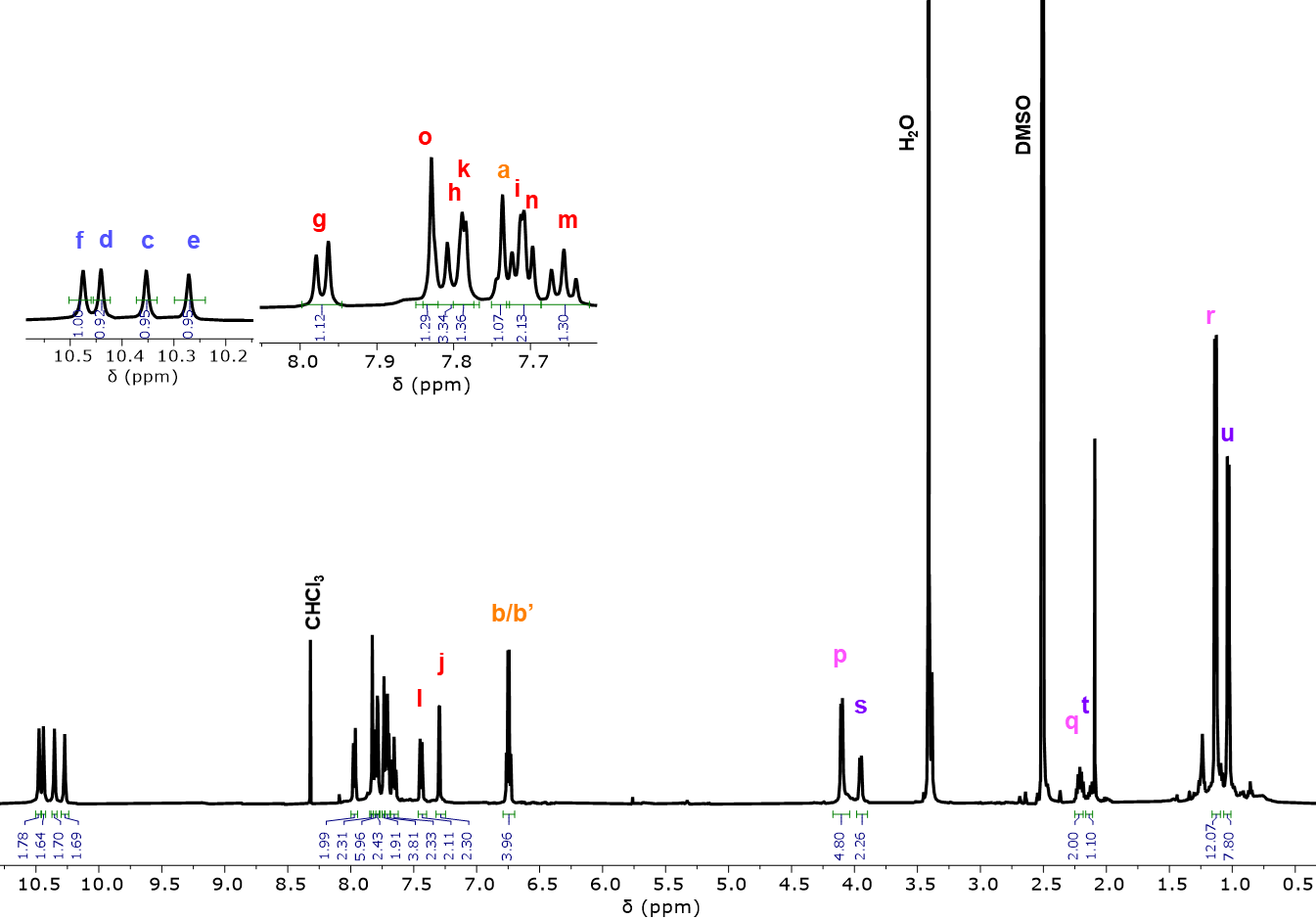


**Figure S1.** Chemical structure of foldamer **B** and detailed ^1^H NMR spectrum of foldamer **B** in DMSO-d_6_.

**Figure S2.** 2D COSY Spectrum of foldamer **B** in DMSO-d_6_.

**Figure S3.** 2D NOESY Spectrum of foldamer **B** in DMSO-d_6_.

**Figure S4.** ^13^C NMR Spectrum of foldamer **B** in DMSO-d_6_

**Figure S5.** HSQC NMR Spectrum of foldamer **B** in DMSO-d_6_

**Figure S6.** HMBC NMR Spectrum of foldamer **B** in DMSO-d_6_


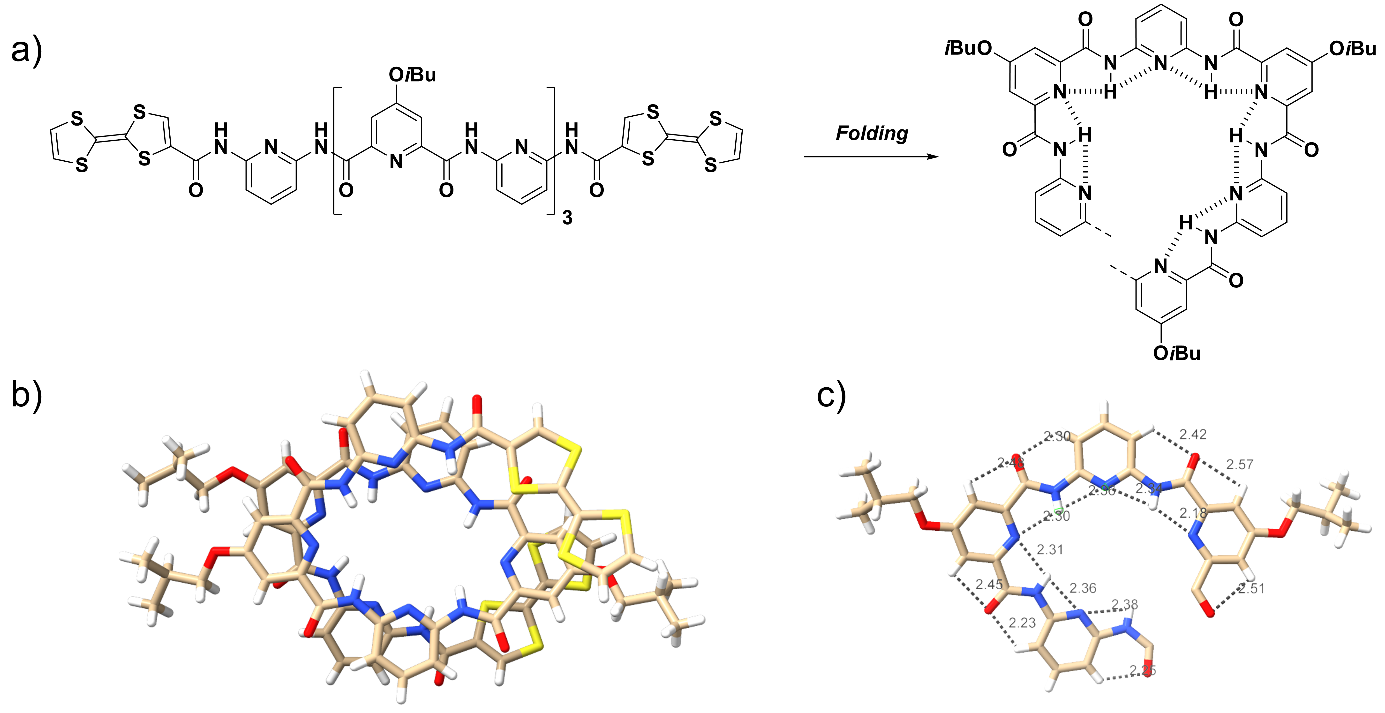


**Figure S7.** a) Schematic representation of foldamer B and its folding process through intramolecular hydrogen bonds. b) Top view of its crystallographic structure (CCDC 2430077, crystals grown by slow diffusion of methanol into a chloroform solution). c) Extract from the crystallographic structure highlighting hydrogen bonds and their lengths (in Ångström).

**
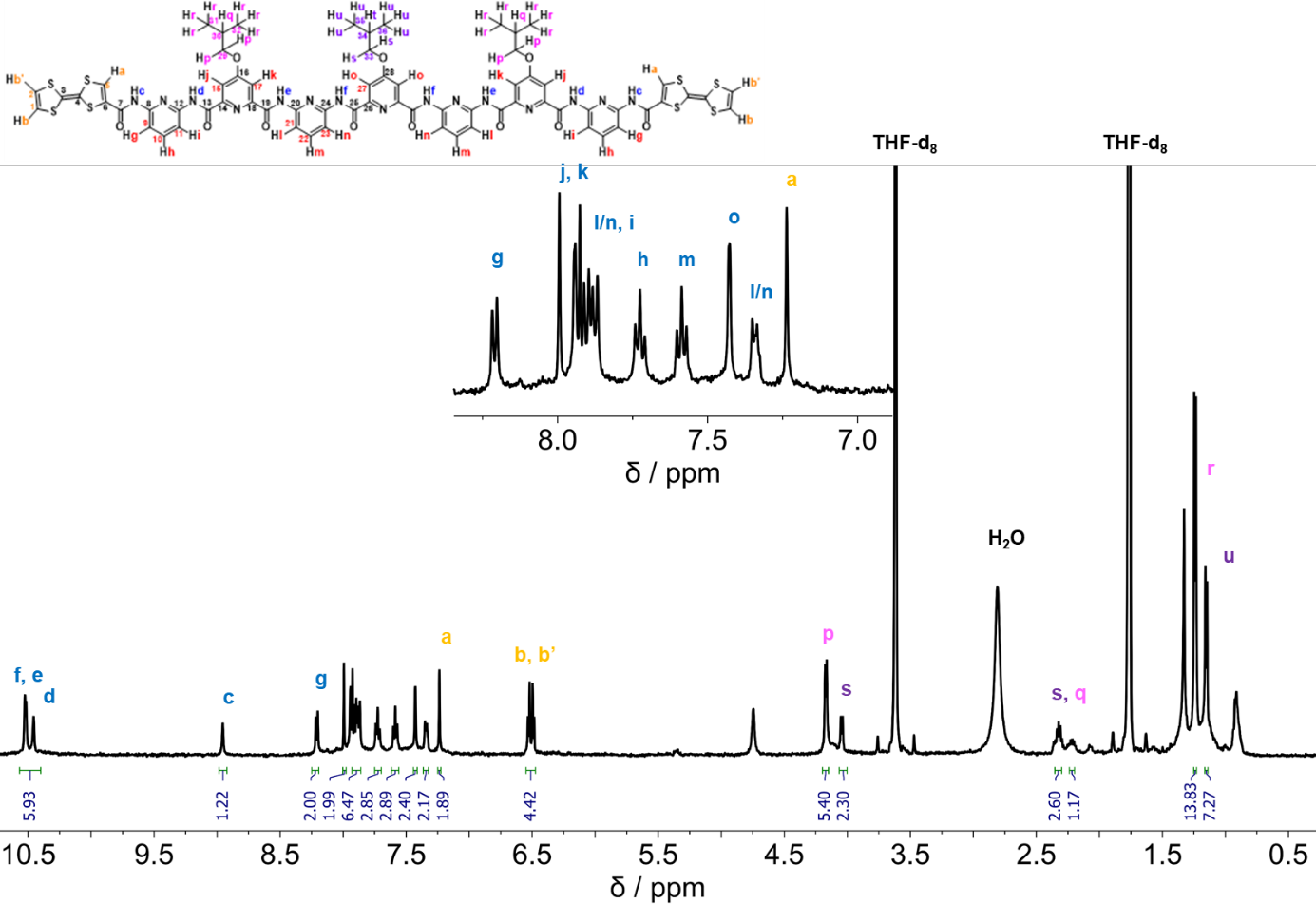
**

**Figure S8.** ^1^H NMR Spectrum of foldamer **B** in THF-d_8_

**Figure S9.** COSY NMR Spectrum of foldamer **B** in THF-d_8_

**Figure S10.** NOESY NMR Spectrum of foldamer **B** in THF-d_8_


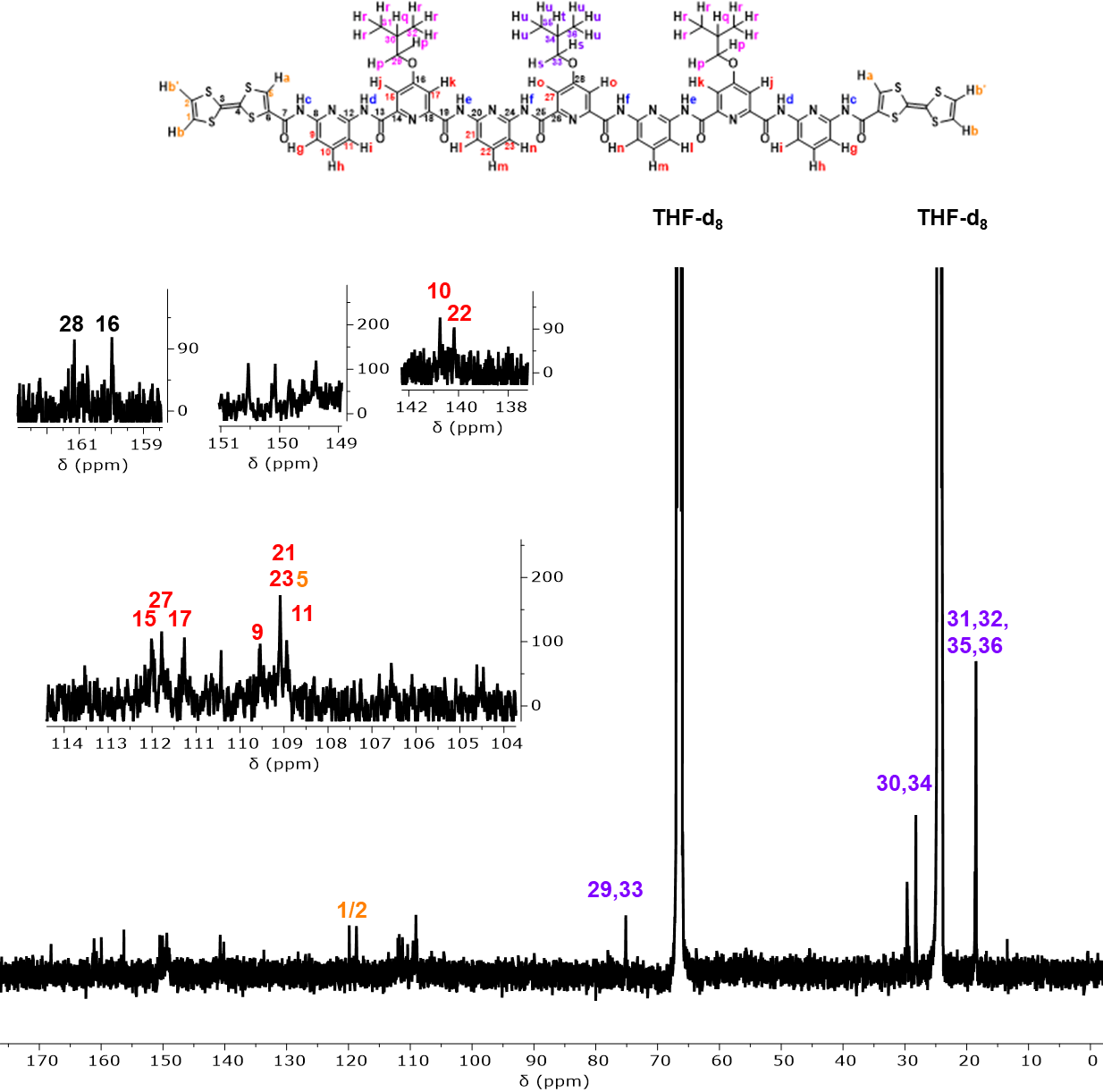


**Figure S11.** ^13^C NMR spectrum of foldamer **B** in THF-d_8_

**Figure S12.** HSQC NMR spectrum of foldamer **B** in THF-d_8_


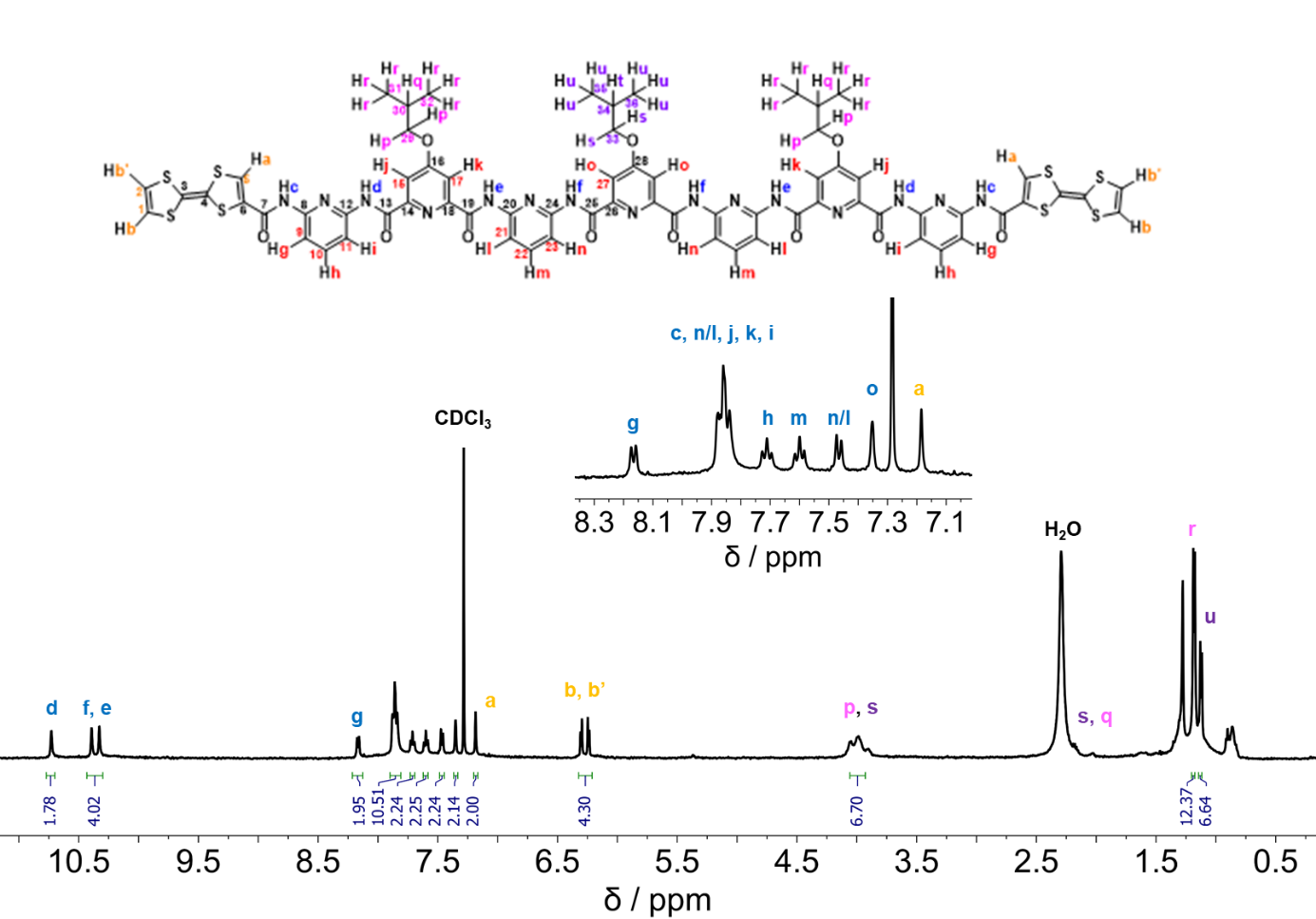


**Figure S13.** ^1^H NMR Spectrum of foldamer **B** in CDCl_3_


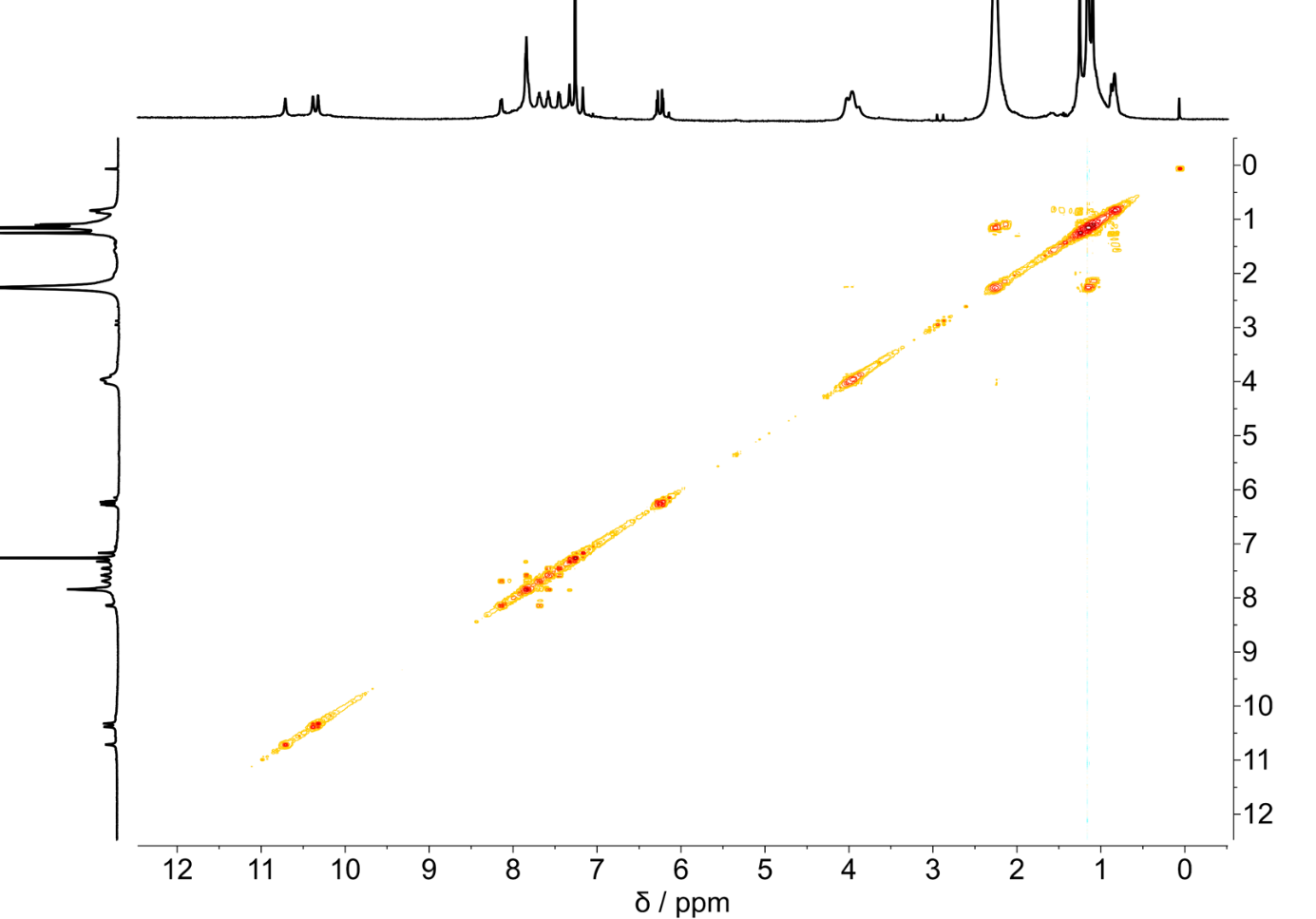


**Figure S14.** COSY NMR Spectrum of foldamer **B** in CDCl_3_


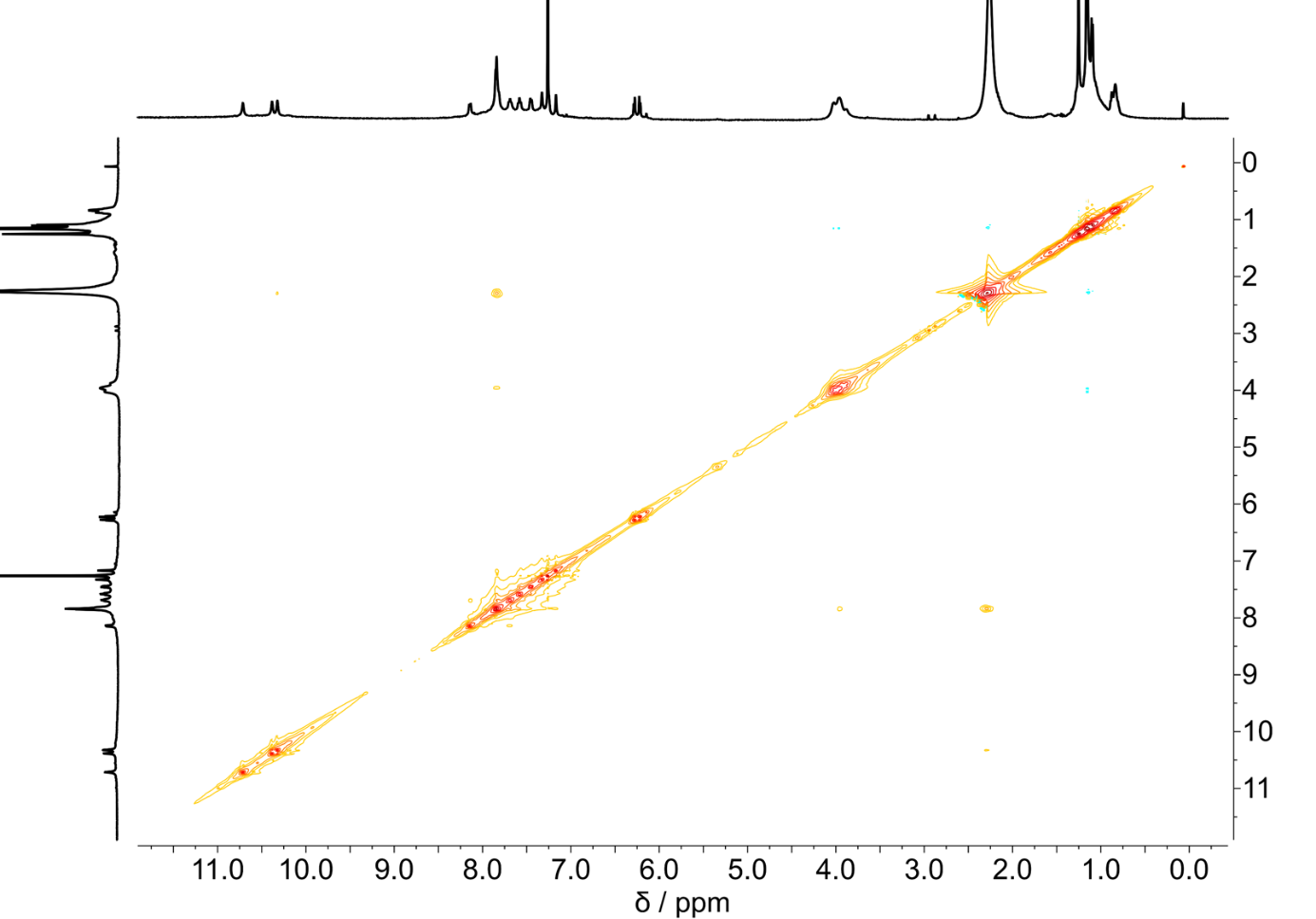


**Figure S15.** NOESY NMR Spectrum of foldamer **B** in CDCl_3_


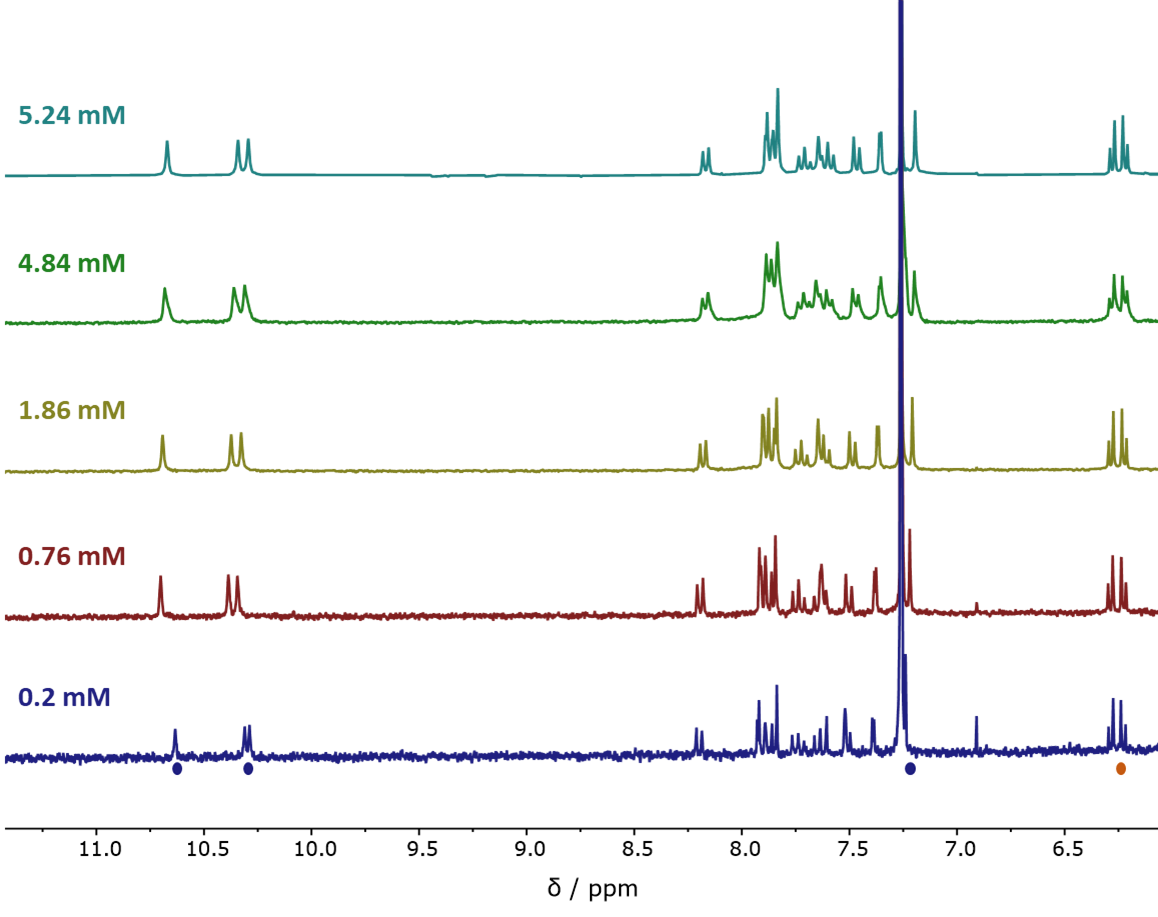


**Figure S16**. ^1^H NMR Spectra of foldamer **B** at different concentrations: blue disks correspond to the amide protons and orange ones correspond to the TTF protons (CDCl_3_, 298 K, 300 MHz).


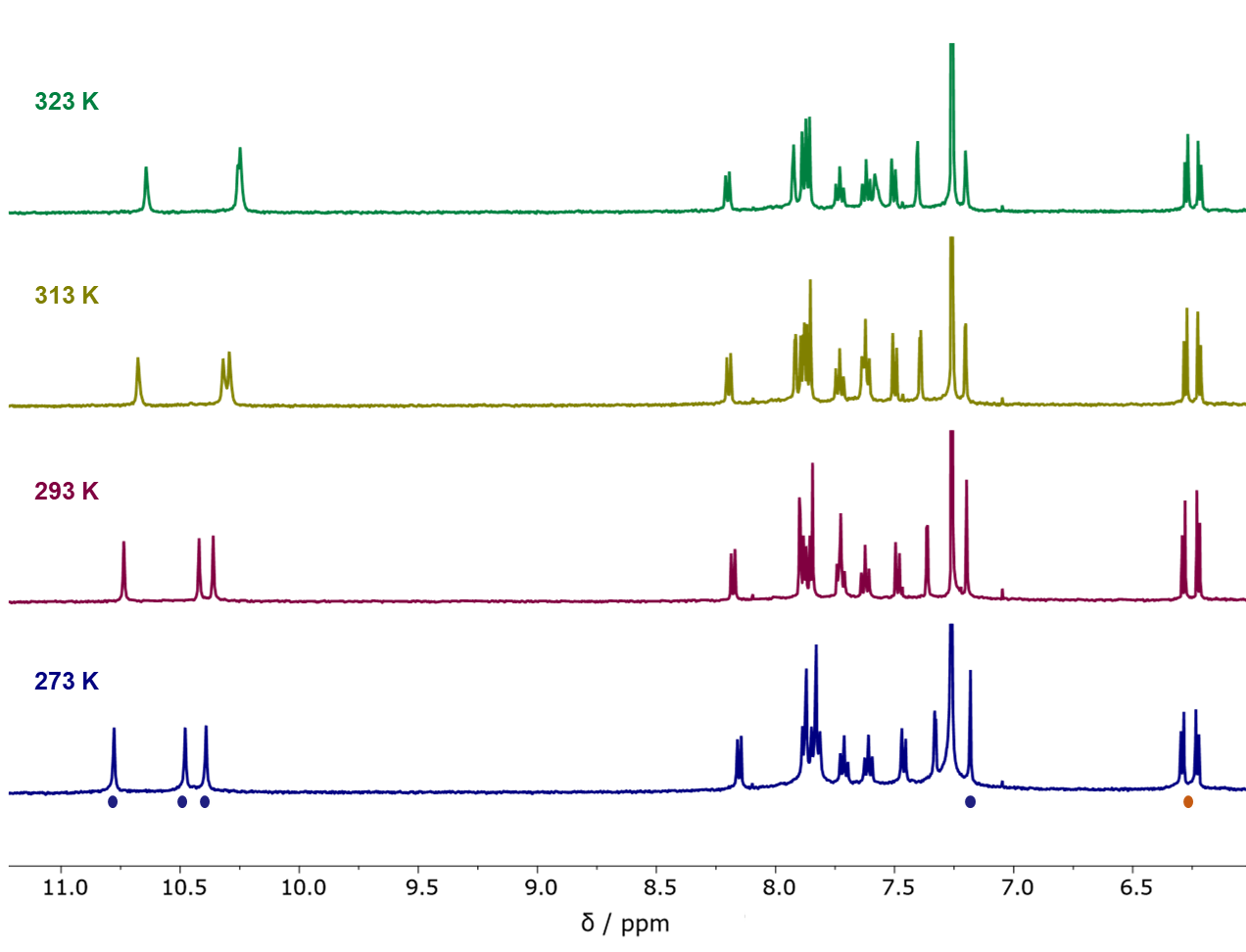


**Figure S17**. ^1^H NMR Spectra of foldamer **A** at different temperatures (5.2 mM, CDCl_3_, 500 MHz).

**Figure S18**. Evolution of the peak intensity i_p_ as a function of the square root of the scan rate recorded during voltammetric studies of foldamer **B**. Pt working electrode, CHCl_3_/ACN (1/1), C = 1.25 × 10^–4^ M, *n*-Bu_4_NPF_6_ (0.1 M).


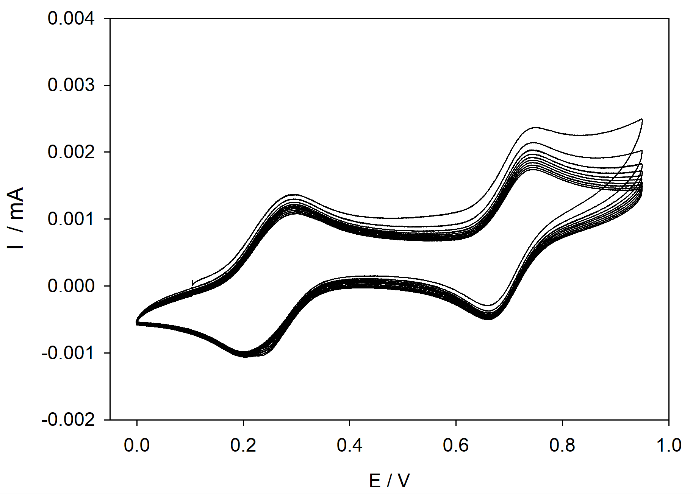


**Figure S19.** Cyclic voltammogram of foldamer **B** over 10 cycles. Pt working electrode, CHCl_3_/ACN (1/1), C = 1.25 × 10^–4^ M, *n*-Bu_4_NPF_6_ (0.1 M), 100 mV.s^-1^.

| 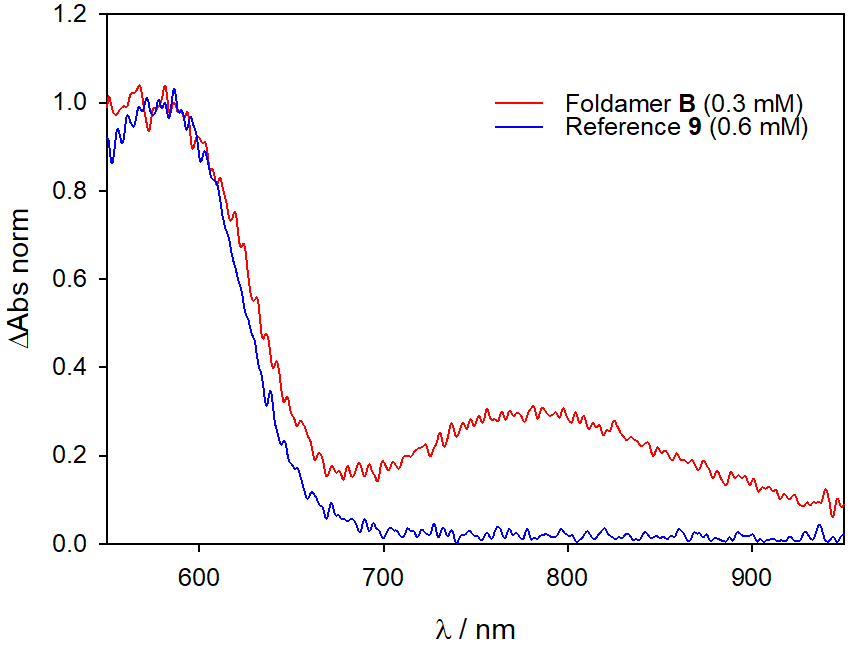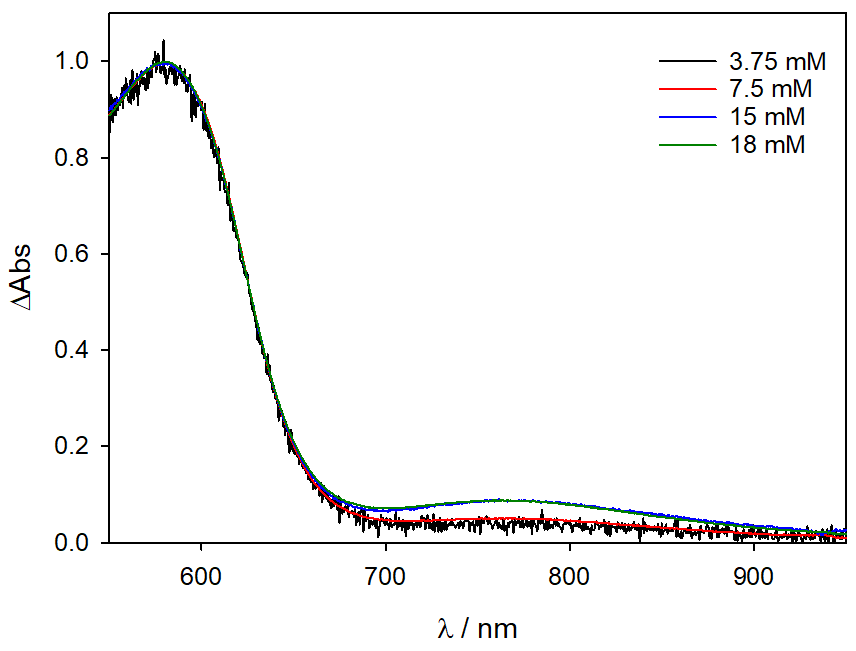 | 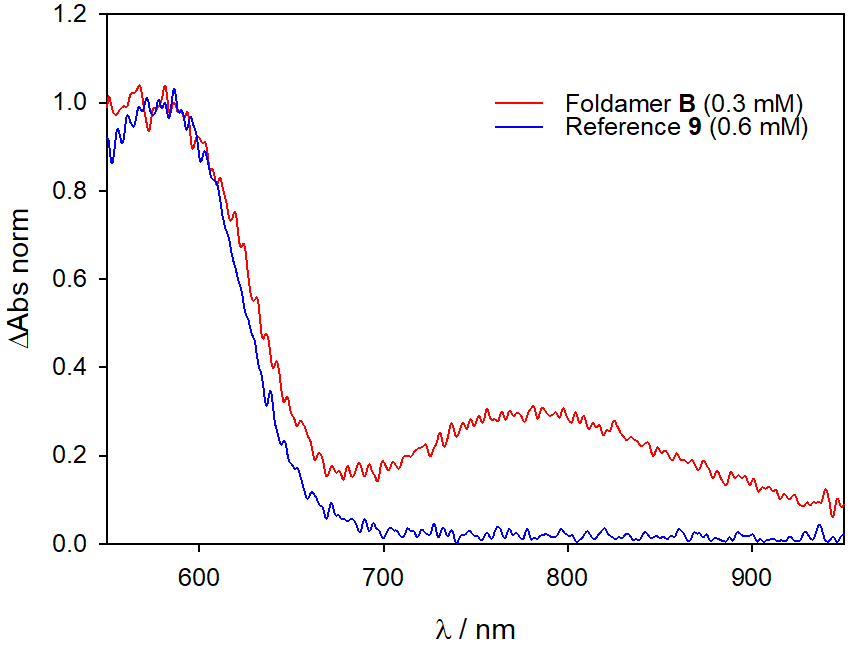 |
| --- | --- |

**Figure S20.** Left. Normalized variations of absorption spectra ΔA(λ) = A_ox_(λ) - A_red_(λ) upon oxidation to the radical cation state for reference compound **9** at different concentrations. Right Normalized variations of absorption spectra upon oxidation to the radical cation state for foldamer **B** (0.3 mM) and **9** (0.6 mM) in CHCl_3_/CH_3_CN 1/1 (v/v).


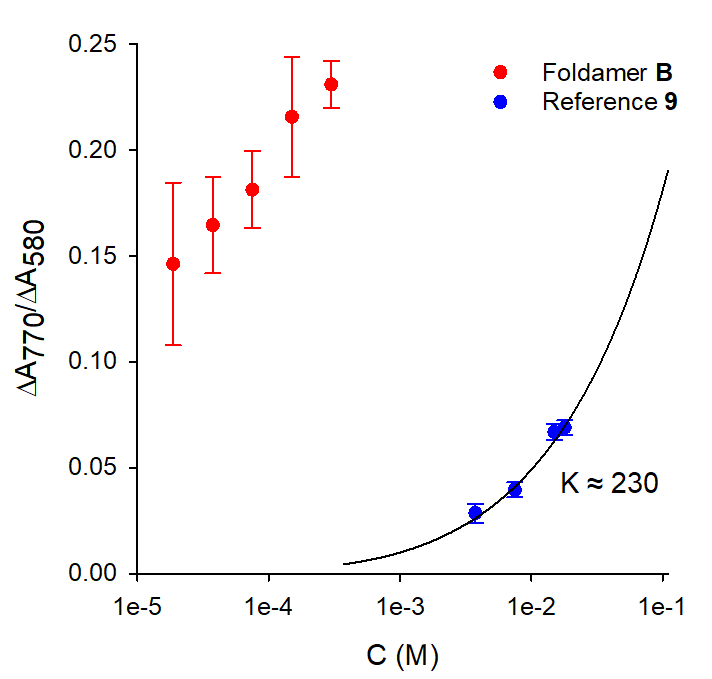


**Figure S21.** Evolutions of the absorbance variation at λ = 770 nm (spectroscopic signature of the π-dimer) for foldamer **B** and reference compound **9** as function of log(C_0_). C_0_ stands for the total concentration.

**Scheme S1.** Chemical structure of a supramolecular macrocycle described by Zhang Ting Li and coworkers,^[26]^ which includes two precursors preorganized through hydrogen bonding.


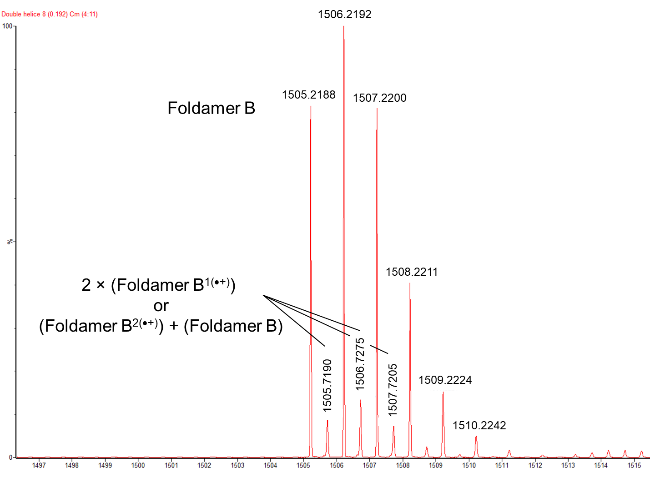

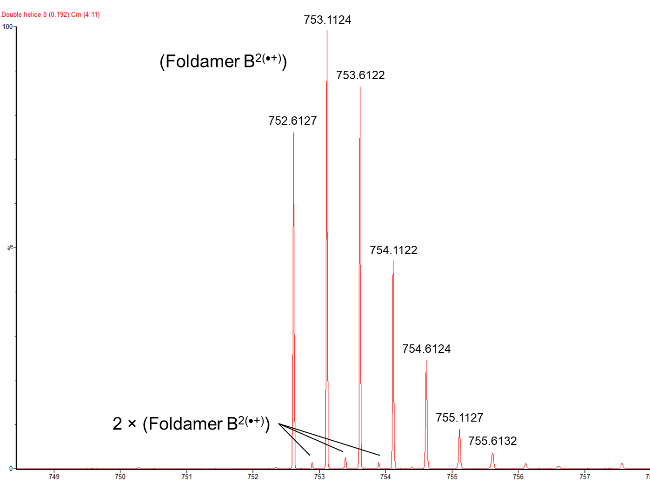


**Figure S22.** ESI Mass spectra recorded of the oxidized duplex (see experimental details for conditions)

**Dimethyl 4-isobutoxypyridine-2,6-dicarboxylate**

Dimethyl 4-*iso*butoxypyridine-2,6-dicarboxylate was synthesized according to the procedure described by Huc and collaborators.^33^

**4-isoButoxypyridine-2,6-dicarboxylic acid 1** (adapted from reference 33)

Potassium hydroxide (800 mg, 14 mmol) was dissolved in methanol (20 mL) and was added to a solution of dimethyl 4-isobutoxypyridine-2,6-dicarboxylate (0.9 g, 2 mmol) in methanol (20 mL). The mixture was stirred at room temperature overnight. A white precipitate appeared, was filtered and washed with 10 mL of methanol. The solid was subsequently suspended in water, protonated with hydrochloric acid 3M, washed with water, and dried under vacuum to afford **2** as a white solid (833 mg, quantitative yield).

**^1^H NMR (300 MHz, DMSO–d_6_)** δ 7.68 (s, 2H), 3.98 (d, J = 6.5 Hz, 2H), 2.05 (m, 1H), 0.98 (d, J = 6.7 Hz, 6H). **^13^C NMR (75 MHz, DMSO–d_6_)** δ 166.9, 165.3, 149.7, 74.5, 27.4, 18.7.

***Tert*-butyl (6-aminopyridin-2-yl)carbamate 2**

*tert*-Butyl (6-aminopyridin-2-yl)carbamate **2** was synthesized according to the procedure described by Woisel and collaborators.^34^

**Compound 3**

Under argon atmosphere, dry diacid **1** (1 g, 4.18 mmol) was mixed with dry dichloromethane (10 mL). Oxalyl chloride (2.65 mg, 20.90 mmol) was added and a drop of dry dimethylformamide. Gas release was observed and the mixture was stirred for two hours under argon atmosphere. Dichloromethane and oxalyl chloride were evaporated under vacuum using a liquid nitrogen trap and the resulting solid was dried under vacuum for three hours and then 8 mL of dry dichloromethane was added. In another Schlenk, dry *tert*-butyl (6-aminopyridin-2-yl)carbamate **2** (1.92 g, 9.20 mmol) was dissolved in 10 mL of dry dichloromethane and dry DIPEA (2.70 g, 20.90 mmol) was added. The resulting mixture was added dropwise to the solution of acid chloride and stirred for 16 hours under argon atmosphere. The solvent was removed under reduced pressure and the crude was purified by silica gel chromatography (eluent dichloromethane:ethyl acetate 99:1) to afford **3** as white powder (1.86 g, 71%).

**^1^H NMR** **(300 MHz, CDCl_3_)** δ 10.18 (s, 2H), 8.08 (dd, J = 7.8, 1.0 Hz, 2H), 7.96 (s, 2H), 7.76 (t, J = 8.0 Hz, 2H), 7.69 (dd, J = 8.2, 1.1 Hz, 2H), 7.24 (s, 2H), 3.96 (d, J = 6.5 Hz, 2H), 2.17 (dq, J = 13.6, 6.8 Hz, 1H), 1.54 (s, 18H), 1.07 (d, J = 6.7 Hz, 6H). **^13^C NMR** **(75 MHz, CDCl_3_)** δ 168.4, 161.8, 152.2, 150.7, 150.5, 149.3, 140.9, 112.3, 109.0, 108.6, 81.4, 28.4, 28.1, 19.1.

**Compound 4** (adapted from reference 14)

Trimer **3** (0.97 g, 1.56 mmol) was dissolved in dichloromethane (8 mL) and trifluoroacetic acid (1.25 g, 10.92 mmol) was added slowly. The mixture was stirred for four hours at room temperature. Then, a saturated solution of sodium bicarbonate (20 mL) and chloroform were added. After extraction, the organic phase was washed three times with water and dried over magnesium sulfate. The solvent was removed under reduced pressure to afford **4** as a white powder (650 mg, quantitative).

**^1^H NMR (300 MHz, CDCl_3_)** δ 10.02 (s, 2H), 7.95 (s, 2H), 7.76 (d, J = 7.9 Hz, 2H), 7.53 (t, J = 7.9 Hz, 2H), 6.30 (d, J = 8.0 Hz, 2H), 4.73 (br, 4H), 3.95 (d, J = 6.5 Hz, 2H), 2.17 (m, 1H), 1.06 (d, J = 6.7 Hz, 6H). **^13^C NMR (75 MHz, CDCl_3_)** δ 168.5, 161.6, 157.7, 150.7, 149.4, 140.5, 112.0, 105.1, 103.8, 75.5, 28.2, 19.2.

**Compound 5**

Amine **5** was synthesized according to the literature from the corresponding carbamate protected analogue.^20^

**Carboxytetrathiafulvalene 6** (adapted from reference 35)

Into a stirred solution of TTF (2 g, 9.8 mmol) in anhydrous diethyl ether (120 mL) at –78°C under argon atmosphere, a solution of LDA 1M in THF/hexane (11 mmol) was added dropwise. A thick yellow suspension appeared and the stirring was continued for 45 min at –78°C. CO_2_ gas was bubbled through the stirred suspension for 1h at –78°C to give an orange suspension and the mixture was then slowly allowed to warm to room temperature over 16 H. The mixture was filtered and the solid was washed thoroughly with diethyl ether, affording the lithium salt of TTF–carboxylate as an orange solid. To a stirred solution of the latter salt in H_2_O, 10 mL of a solution of hydrochloric acid 1M were added slowly and a deep red solid precipitated immediately. The red solid was collected by filtration, washed thoroughly with water and dried under vacuum to give **9** (2.2 g, 88 %).

**^1^H NMR (300 MHz, Acetone–d_6_)** δ 7.63 (s, 1H), 6.66 (s, 2H).

**Compound 7**

To a solution of TTF-COOH **6** (530 mg, 2.13 mmol) in 15 mL dry dichloromethane under argon atmosphere at 0°C, 4-dimethylaminopyridine (111 mg, 0.914 mmol) was added. The mixture was stirred at 0°C for 10 minutes and then, N,N’-dicyclohexylcarbodiimide (314 mg, 1.52 mmol) was added. The mixture was stirred for 40 minutes at 0°C. In another Schlenk, amine **5** (524 mg, 1.52 mmol) was dissolved in 15 mL of dry dichloromethane and the resulting solution was added dropwise to the solution of activated acid at 0°C. The mixture was stirred overnight at room temperature under argon atmosphere. The resulting suspension was filtrated and the filtrate was purified by silica gel chromatography (eluent dichloromethane: ethyl acetate: triethylamine 95:4:1) to afford compound **7** as a red solid (440 mg, 53%).

**^1^H NMR (500 MHz, DMSO–d_6_)** δ 11.11 (s, 1H), 10.23 (s, 1H), 8.12 (s, 1H), 8.05 (d, J = 8.0 Hz, 1H), 7.94 (t, J = 8.1 Hz, 1H), 7.89 (d, J = 2.4 Hz, 1H), 7.84 (d, J = 8.2 Hz, 1H), 7.79 (d, J = 2.4 Hz, 1H), 6.76 (s, 2H), 4.06 (d, J = 6.5 Hz, 2H), 3.95 (s, 3H), 2.08 (s, 1H), 1.02 (d, J = 6.7 Hz, 6H). **^13^C NMR (125 MHz, DMSO–d_6_)** δ 167.5, 164.0, 161.0, 150.4, 148.8, 141.0, 133.2, 128.5, 120.3, 120.0, 114.7, 113.0, 111.1, 110.4, 105.2, 74.8, 52.9, 30.6, 27.4, 18.7. **HRMS** (MALDI–TOF) calcd. for C_67_H_59_N_15_O_11_S_8_ [M]^+^ ; 574.0461; found, 574.0467.

**Compound 8**

Solid lithium hydroxide (45 mg, 1.9 mmol) was dissolved in a minimal amount of water and was added dropwise to a solution of **7** (440 mg, 0.7 mmol) in 20 mL of tetrahydrofuran. The mixture was stirred at room temperature for three hours. Then, acetic acid was added dropwise to a neutral pH (monitored by litmus paper). The solvent was evaporated under reduced pressure and the resulting solid was washed by water and filtrated to afford compound **8** as a red powder (430 mg, quantitative).

**^1^H NMR (500 MHz, DMSO–d_6_)** δ 11.11 (s, 1H), 10.23 (s, 1H), 8.12 (s, 1H), 8.05 (d, J = 8.0 Hz, 1H), 7.94 (t, J = 8.1 Hz, 1H), 7.89 (d, J = 2.4 Hz, 1H), 7.84 (d, J = 8.2 Hz, 1H), 7.79 (d, J = 2.4 Hz, 1H), 6.76 (s, 2H), 4.06 (d, J = 6.5 Hz, 2H), 2.08 (s, 1H), 1.02 (d, J = 6.7 Hz, 6H). **^13^C NMR (125 MHz, DMSO–d_6_)** δ 166.7, 166.7, 161.9, 157.8, 150.2, 149.3, 149.1, 140.9, 133.4, 128.3, 120.2, 120.0, 112.9, 109.8, 108.9, 108.6, 105.2, 74.2, 45.3, 27.4, 18.8, 9.0. **HRMS** (MALDI–TOF) calcd. for C_23_H_20_N_4_O_5_S_4_ [M]^+^ ; 560.0317; found, 560.0322.

**Foldamer B**

Dry acid **8** (98 mg, 0.2 mmol, 2.3 equivalents) was dissolved in dry dichloromethane (5 mL) under argon atmosphere and 1–chloro–*N*,*N*,2–trimethyl–1–propenylamine (Ghosez’s reagent, 61 mg, 0.45 mmol, 6 equivalents) was added. The mixture was stirred for 4 hours under argon atmosphere. The latter was evaporated under vacuum using a liquid nitrogen trap. The resulting solid was dried for 3 hours under vacuum and 6 mL of dry tetrahydrofuran was added. In another Schlenk, dry **4** (32 mg, 0.7 mmol) was dissolved in 4 mL of dry tetrahydrofuran and dry DIPEA (52 mg, 0.6 mmol) was added. The latter mixture was added dropwise to the solution of acyl chloride and was stirred for 20 hours at room temperature. The solvent was removed under reduced pressure and the crude was purified by silica gel chromatography (eluent DCM/EtOAc/Et_3_N 79/20/1) and subsequently, recycling HPLC to afford foldamer **B** as an orange–red solid (42 mg, 37 %).

**^1^H NMR (500 MHz, DMSO–d_6_)** δ 10.47 (s, 2H), 10.43 (s, 2H), 10.35 (s, 2H), 10.26 (s, 2H), 7.96 (d, J = 7.9 Hz, 2H), 7.82 (d, J = 2.5 Hz, 2H), 7.80 (s, 2H), 7.79 – 7.77 (m, 2H), 7.73 (d, J = 4.2 Hz, 2H), 7.72 – 7.69 (m, 4H), 7.65 (t, J = 8.0 Hz, 2H), 7.43 (d, J = 7.9 Hz, 2H), 7.29 (d, J = 2.4 Hz, 2H), 6.74 (q, J = 6.3 Hz, 4H), 4.09 (d, J = 6.5 Hz, 4H), 3.94 (d, J = 6.8 Hz, 2H), 2.20 (m, 2H), 2.14 – 2.10 (m, 1H), 1.13 (d, J = 6.7 Hz, 12H), 1.03 (d, J = 6.7 Hz, 6H). **^13^C NMR (125 MHz, DMSO–d_6_)** δ 168.8, 168.5, 161.6, 161.3, 160.7, 157.6, 150.8, 150.7, 150.4, 149.6, 149.5, 149.1, 141.9, 141.3, 134.3, 128.7, 121.3, 120.8, 112.6, 112.3, 112.0, 111.7, 110.3, 109.9, 109.7, 109.64, 106.40, 80.1, 75.8, 31.6, 28.5, 28.4, 19.8, 19.7. **HRMS** (MALDI–TOF) calcd. for C_67_H_59_N_15_O_11_S_8_ [M + Na]^+^ ; 1528.2160; found, 1528.2176.

**Reference 9**

To a solution of **6** (132 mg, 535 µmol, 1.4 eq.) in 15 mL dry dichloromethane under argon atmosphere at 0°C, 4–dimethylaminopyridine (65 mg, 321 µmol, 0.6 eq.) was added. The mixture was stirred at 0°C for 10 minutes and then, *N,N’*–dicyclohexylcarbodiimide (110 mg, 535 µmol, 1 eq.) was added. The mixture was stirred for 40 minutes at 0°C. In another Schlenk, **2** (80 mg, 382 µmol) was dissolved in 15 mL of dry dichloromethane and the resulting solution was added dropwise to the solution of activated acid at 0°C. The mixture was stirred overnight at room temperature under argon atmosphere. The resulting suspension was filtrated and the filtrate was purified by silica gel chromatography (eluent DCM/EtOAc/Et_3_N 95/4/1) to afford compound **9** as a red solid (89 mg, 53 %).

**^1^H NMR (300 MHz, DMSO–d_6_)** δ 10.54 (s, 1H), 9.43 (s, 1H), 8.03 (s, 1H), 7.73 (t, J = 8.1 Hz, 1H), 7.51 (ddd, J = 14.9, 8.1, 0.8 Hz, 2H), 6.74 (d, J = 6.6 Hz, 2H), 1.47 (s, 9H). **^13^C NMR (75 MHz, DMSO–d_6_)** δ 157.8, 149.5, 139.9, 133.3, 128.1, 120.3, 120.0, 112.7, 108.9, 105.4, 79.8, 28.0. **HRMS** (MALDI–TOF) calcd. for C17H17N3O3S4 [M]+ ; 439.0153; found, 439.0147.

**Collection of spectra**


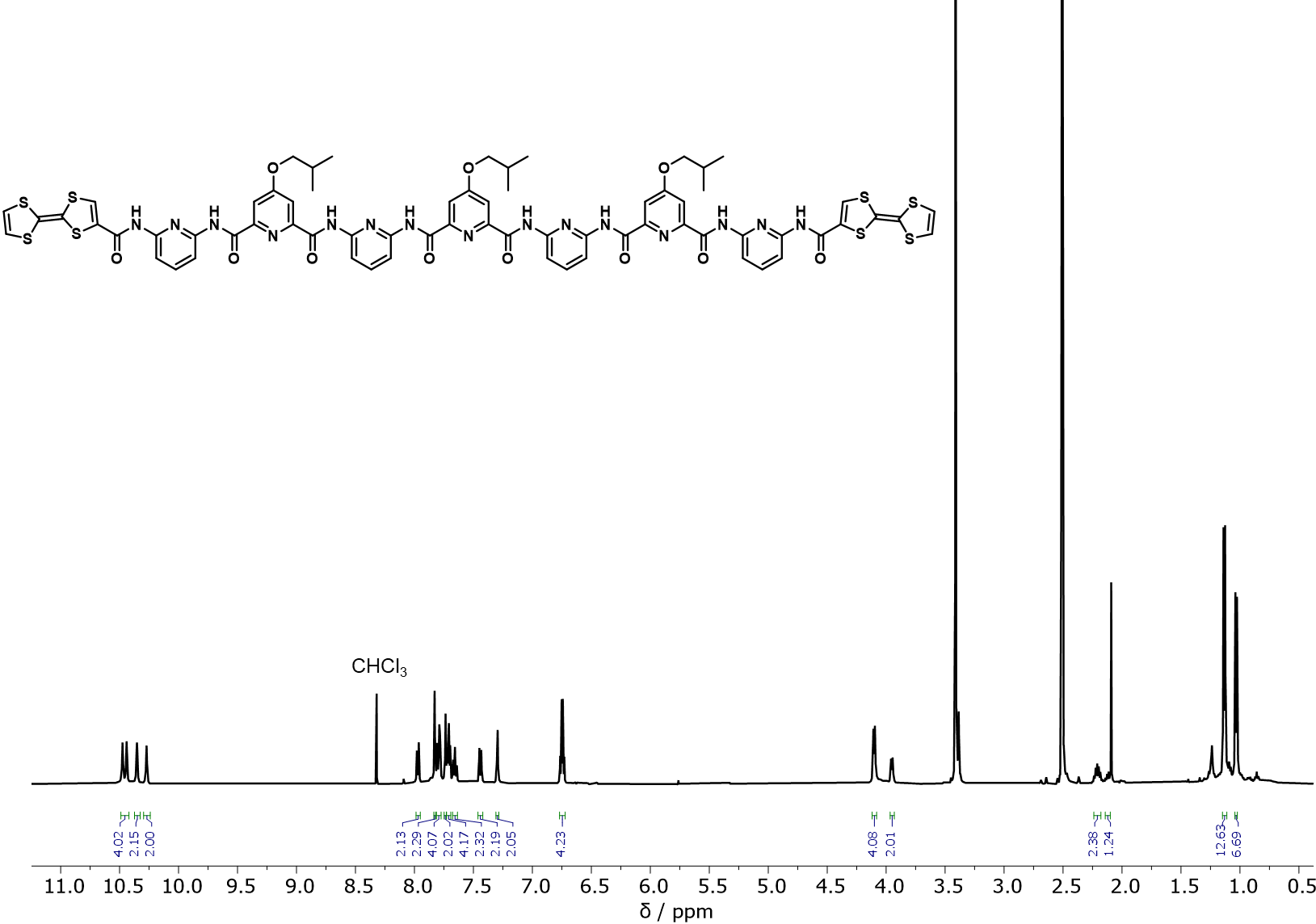


**Figure S23**. ^1^H NMR Spectrum of foldamer **B** in DMSO-d_6_ at room temperature.

**Figure S24**. ^13^C NMR Spectrum of foldamer **B** in DMSO-d_6_ at room temperature.


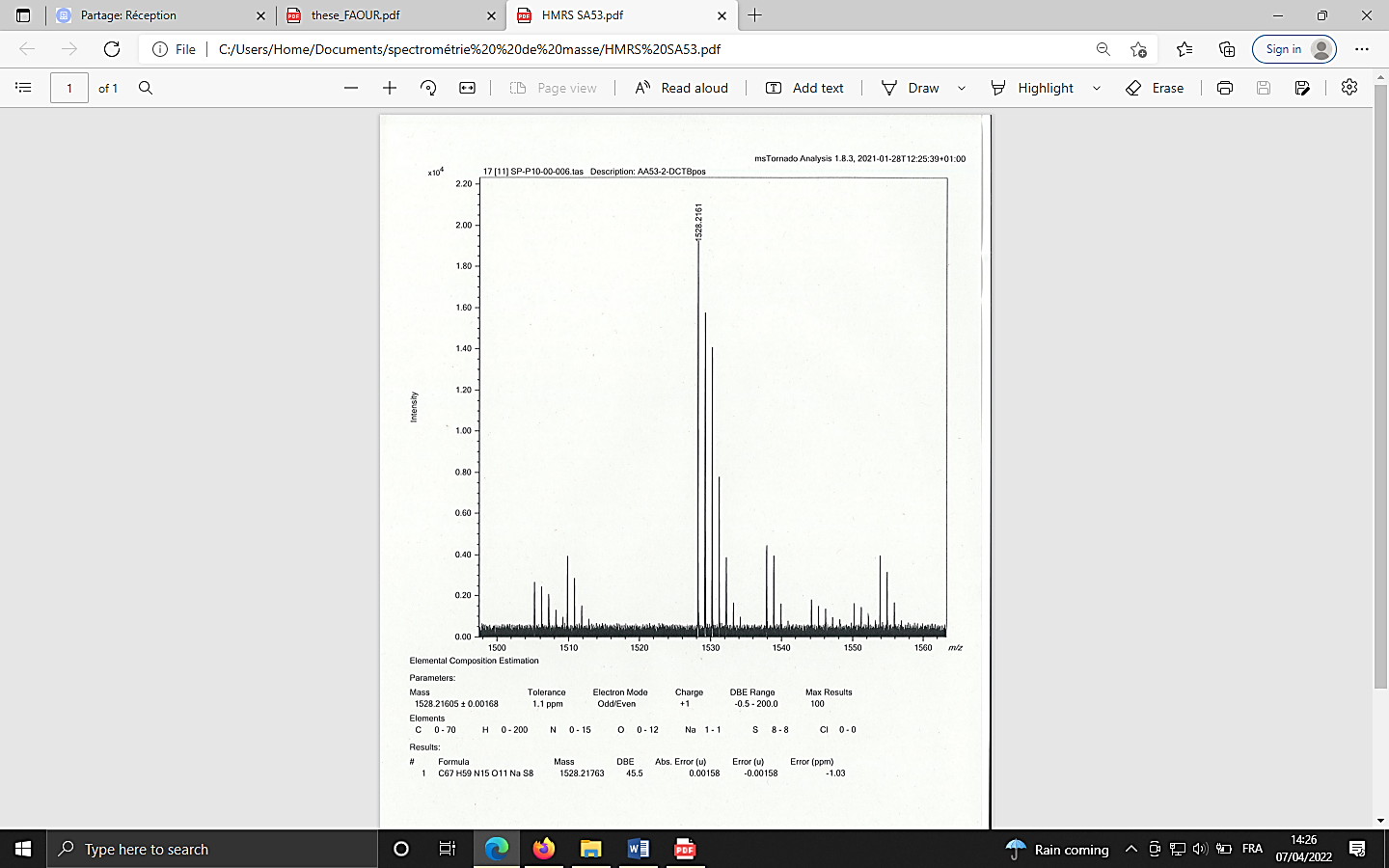


**Figure S25**. HRMS Spectrum of foldamer **B**.

**Compound 7**

**
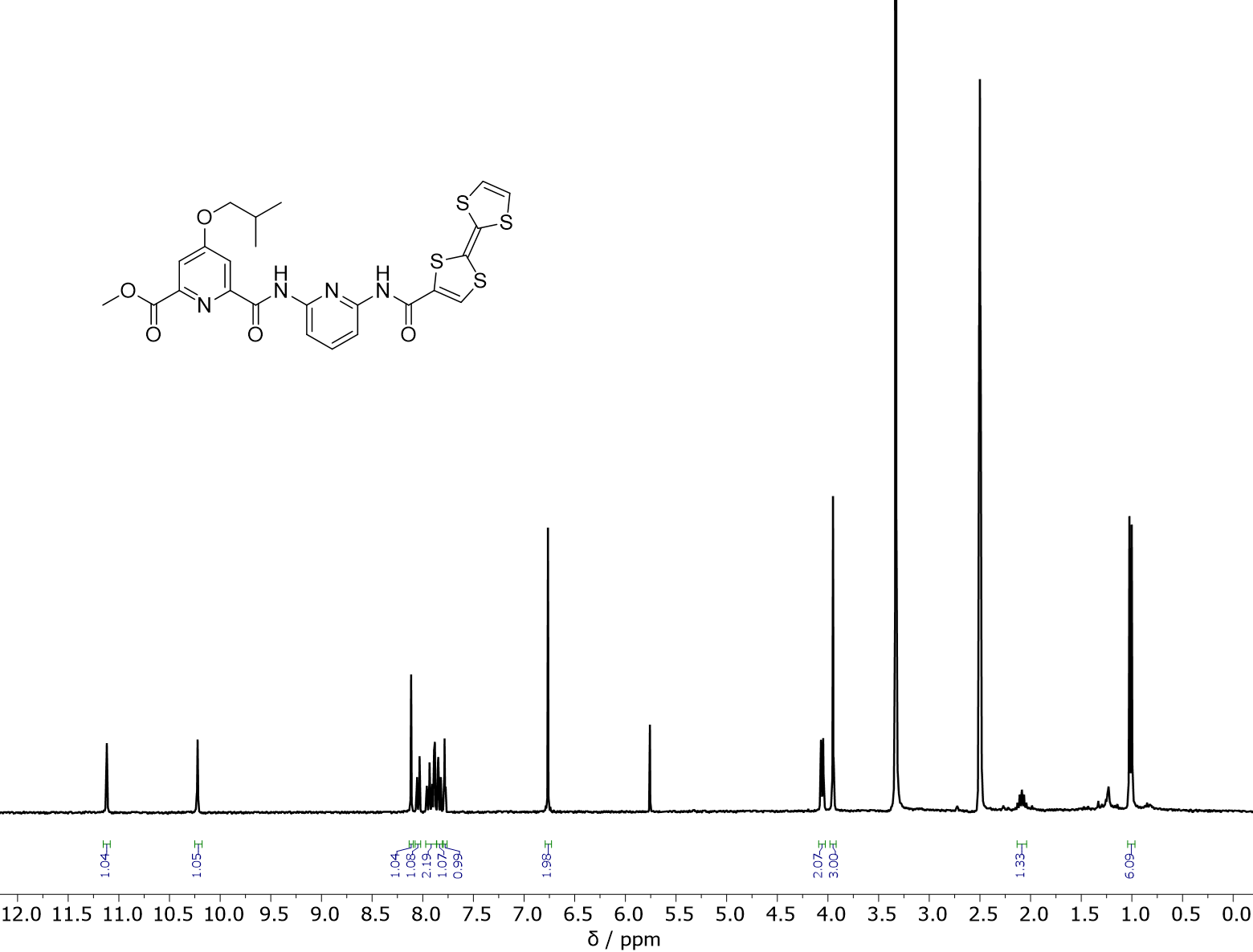
**

**Figure S26.** ^1^H NMR Spectrum of compound **7** in DMSO-d_6_ at room temperature.

**Figure S27**. ^13^C NMR Spectrum of compound **7** in DMSO-d_6_ at room temperature.


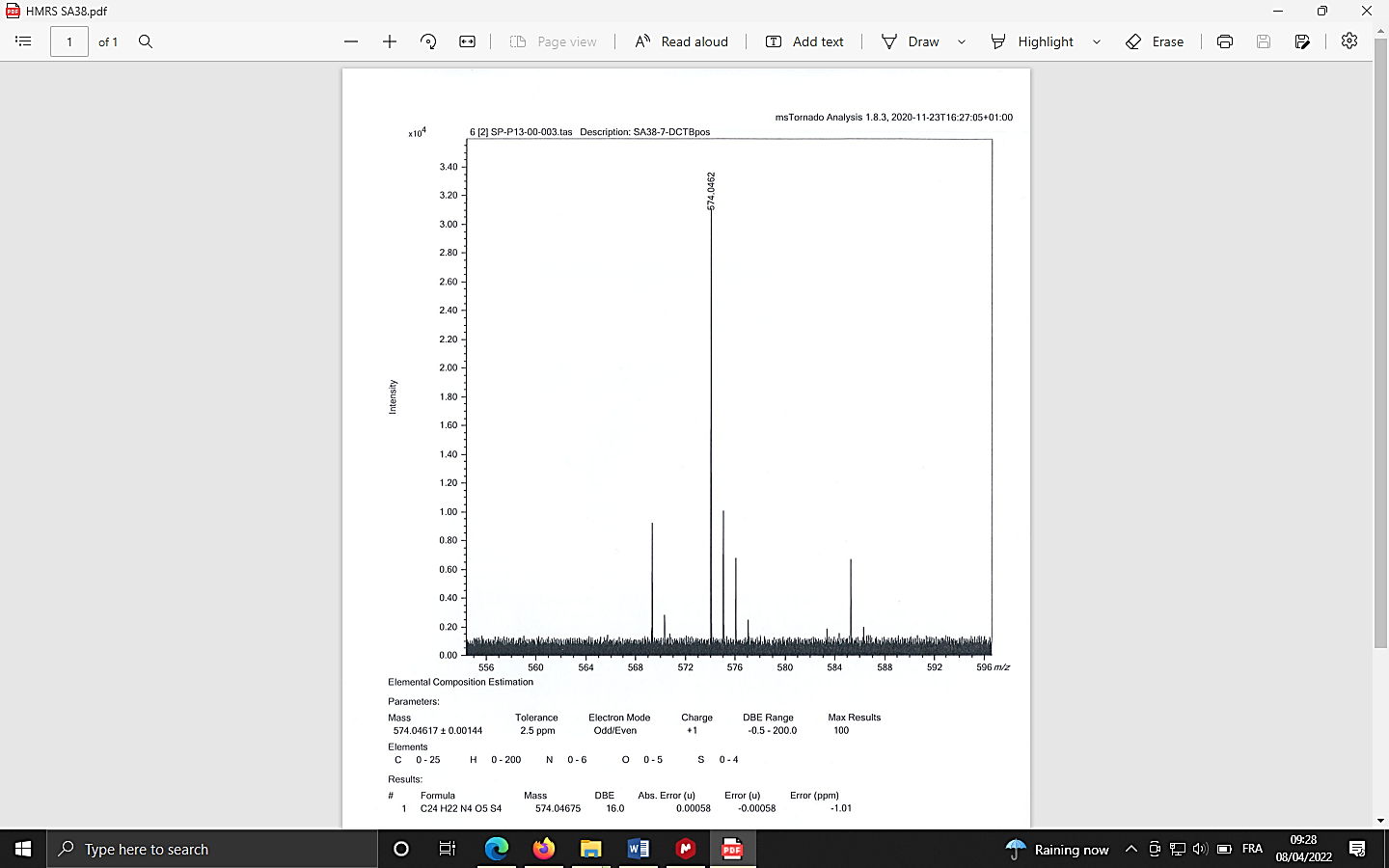


**Figure S28**. HRMS Spectrum of compound **7**.

**Compound 8**

**
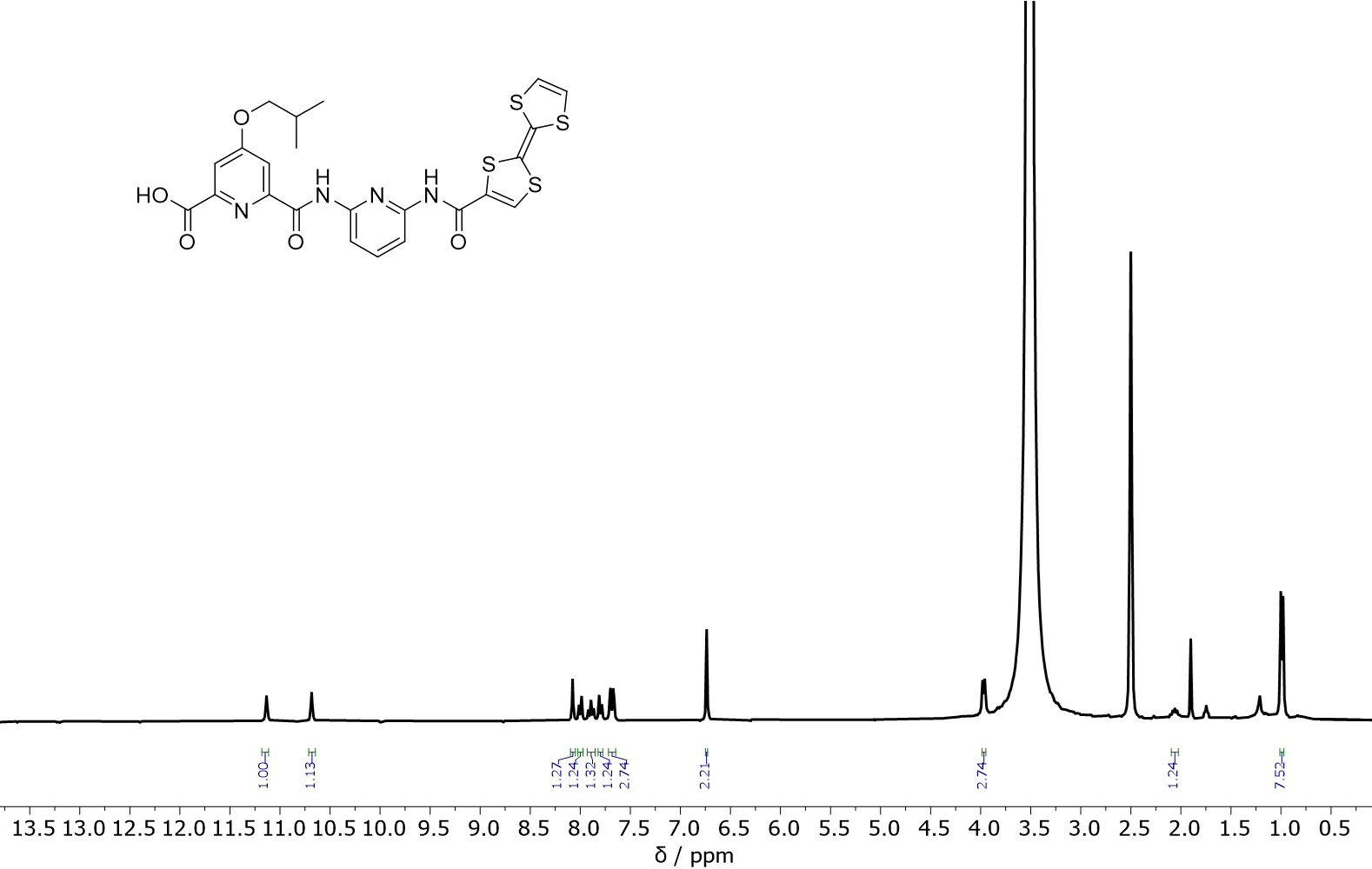
**

**Figure S29.** ^1^H NMR Spectrum of compound **8** in DMSO-d_6_ at room temperature.

**Figure S30**. ^13^C NMR Spectrum of compound **8** in DMSO-d_6_ at room temperature.


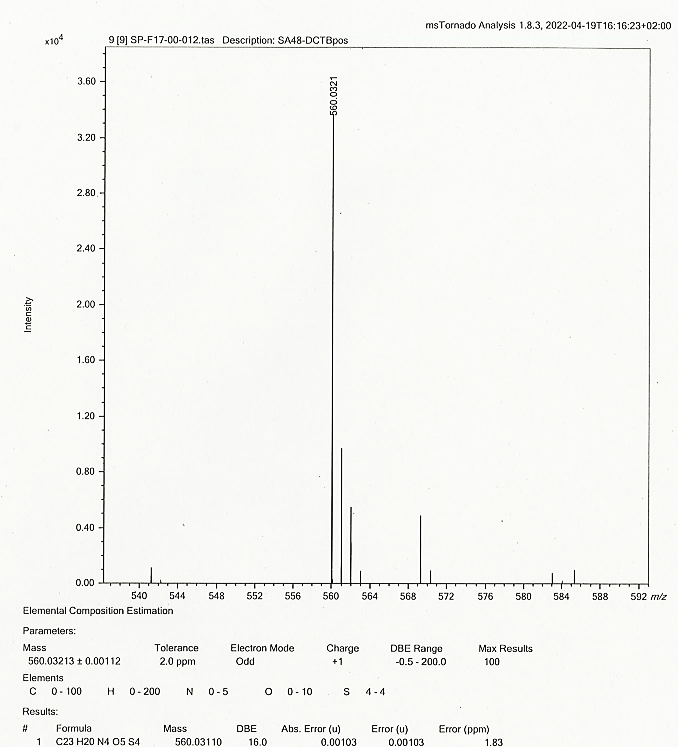


**Figure S31**. HRMS Spectrum of compound **8**.

**Compound 9**

**
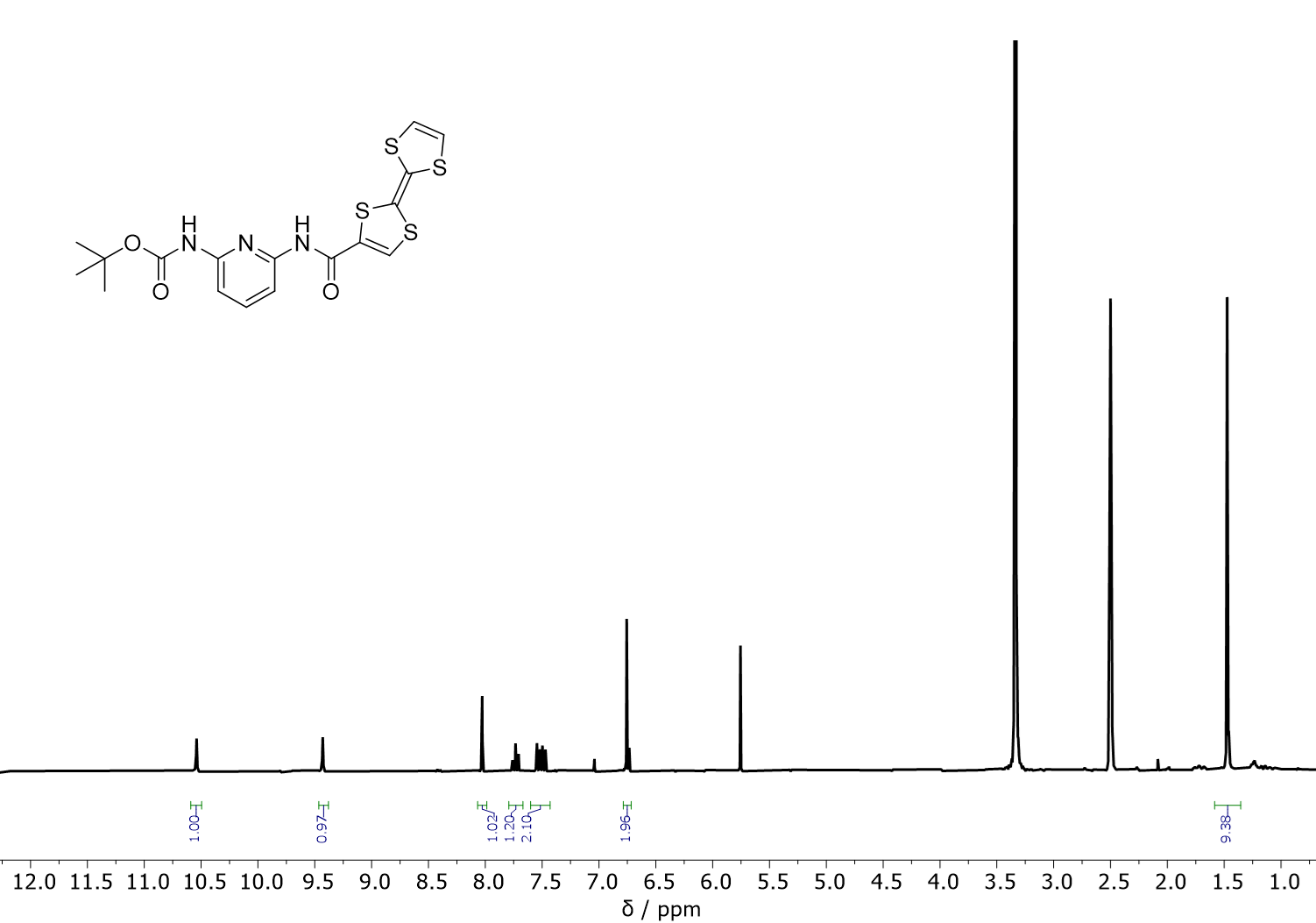
**

**Figure S32.** ^1^H NMR Spectrum of compound **9** in DMSO-d_6_ at room temperature.

**Figure S33**. ^13^C NMR Spectrum of compound **9** in DMSO-d_6_ at room temperature.


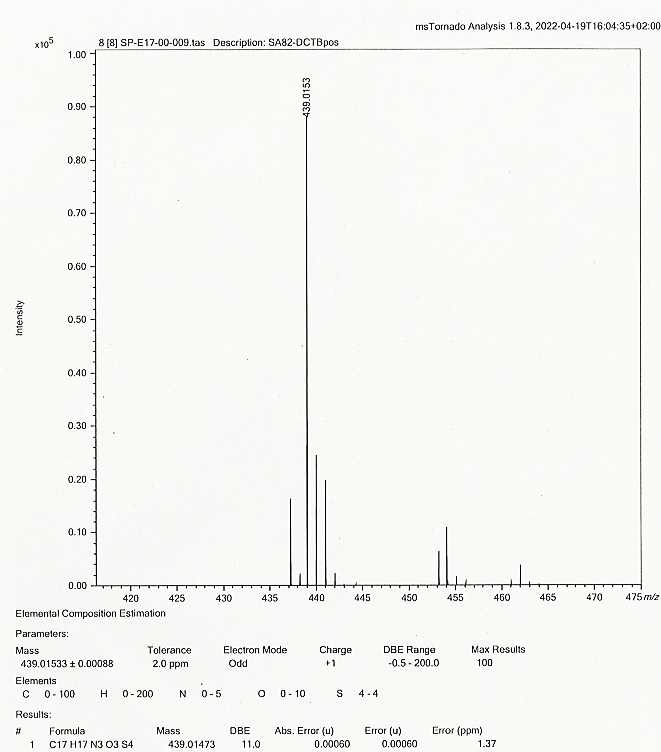


**Figure S34**. HRMS Spectrum of compound **9**.
